# Supplementary material for: Reversal of biological age in multiple rat organs by young porcine plasma fraction
Source: GeroScience. 2023 Oct 24;46(1):367–94. doi: 10.1007/s11357-023-00980-6 (PMC10828479; doi:10.1007/s11357-023-00980-6)
Supplement: Supplementary file 1 — (DOCX 20390 kb) [file 11357_2023_980_MOESM1_ESM.docx]

**Supplementary Material**

for the article “**Reversal of Biological Age in Multiple Rat Organs by Young Porcine Plasma Fraction**”

by S. Horvath et al. Harold Katcher

The Supplement contains Supplementary Figures, Supplementary Tables, Supplementary Methods, and software code for the rat clocks.

**Supplementary Figure 1. Overview of the four distinct rat studies conducted to assess the E5 rejuvenation treatment.** Study 1 underlies Figure 1. Study 2 underlies Figure 2, Figure 3, Figure 4, Figure 5, Figure 6. Study 3 underlies Supplementary Figure 14, Supplementary Figure 15. Study 4 underlies Figure 7.

**Supplementary Figure 2. Unsupervised Hierarchical Clustering of Rat Tissue Samples Based on Inter-array Correlations**. This figure visualizes the unsupervised hierarchical clustering of rat tissue samples, generated using average linkage clustering based on 1 minus the Pearson correlation as dissimilarity measure (utilizing the R hClust function). Five color-coded bands below the dendrogram provide additional information: The first band denotes the cluster branch, indicating the grouping of samples. The second band specifies tissue types, represented as blue for brain, yellow for hypothalamus, turquoise for blood, green for liver, and brown for heart. The third band identifies the test data utilized to evaluate the E5 treatment, marked in blue. The fourth band signifies samples that underwent the E5 treatment, represented in red. The fifth band differentiates age groups: white symbolizes young samples, while red stands for older ones. Notably, the branching within the dendrogram closely aligns with tissue types.


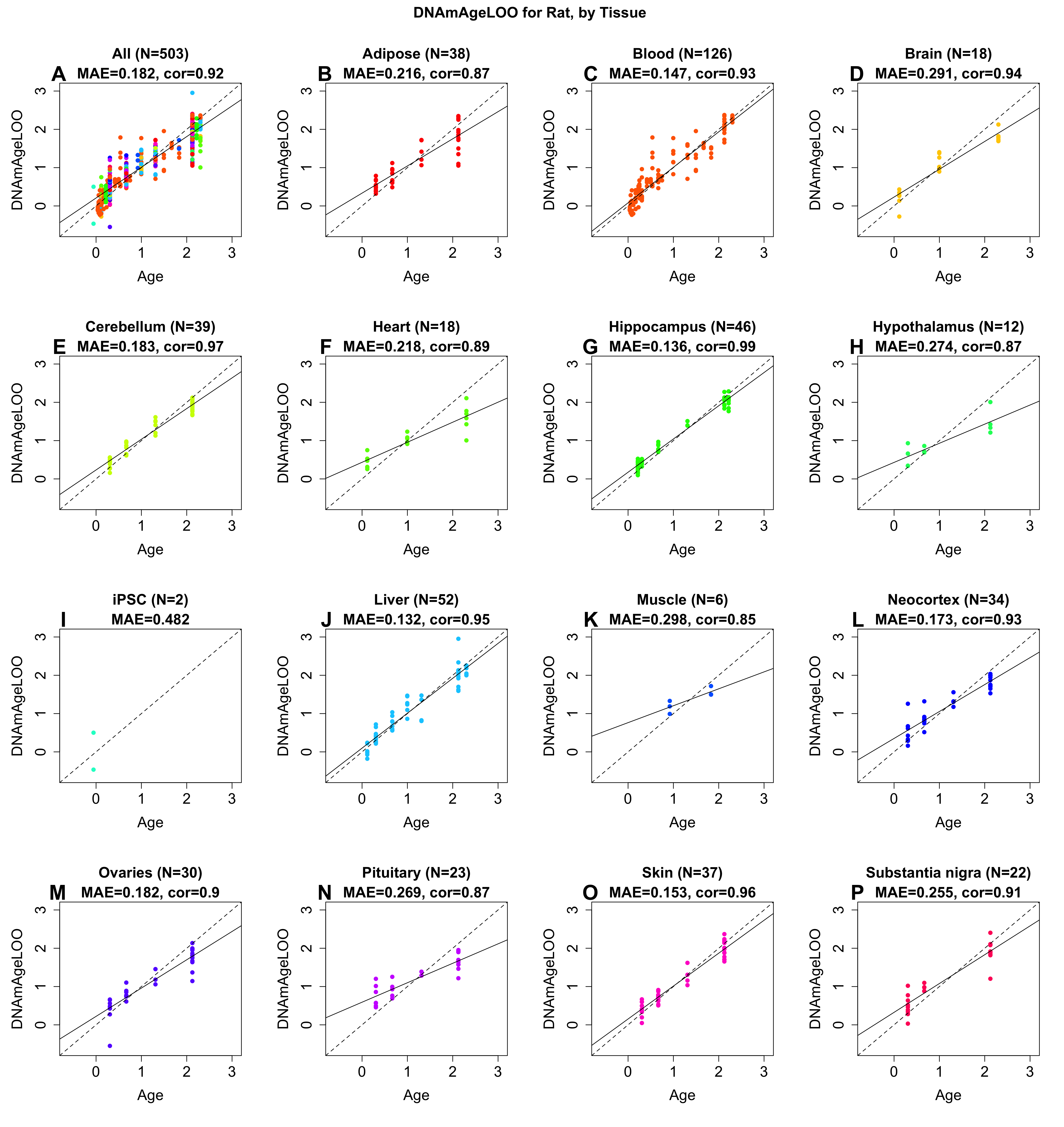


**Supplementary Figure 3. Pan tissue clock for rats applied to different tissues**. A) All tissues. B) adipose, C) blood, D) whole brain, E) cerebellum, F) heart, G) hippocampus, H) hypothalamus, I) liver, J) brain neocortex, K) ovaries, L) pituitary, M) skin, N) substantia nigra. The title of each panel reports the tissue, sample size, Pearson correlation coefficient and median absolute deviation (median error). Chronological age (x-axis) versus leave-one-sample-out estimates of age.

**Supplementary Figure 4: Epigenetic clocks applied to independent test data.** The six epigenetic clocks were applied to DNA profiles from un-treated samples from the E5 plasma fraction study. The y-axis reports epigenetic age (in years) as measured by the indicated clocks. The heading reports the tissue type, the correlation between epigenetic and chronological age and the median error (in years). While the age correlations are high, the median errors are sub-optimal, up to 0.38 years (panel J). As such, the final versions of the clocks incorporated un-treated samples from the test data as well (Methods).

**
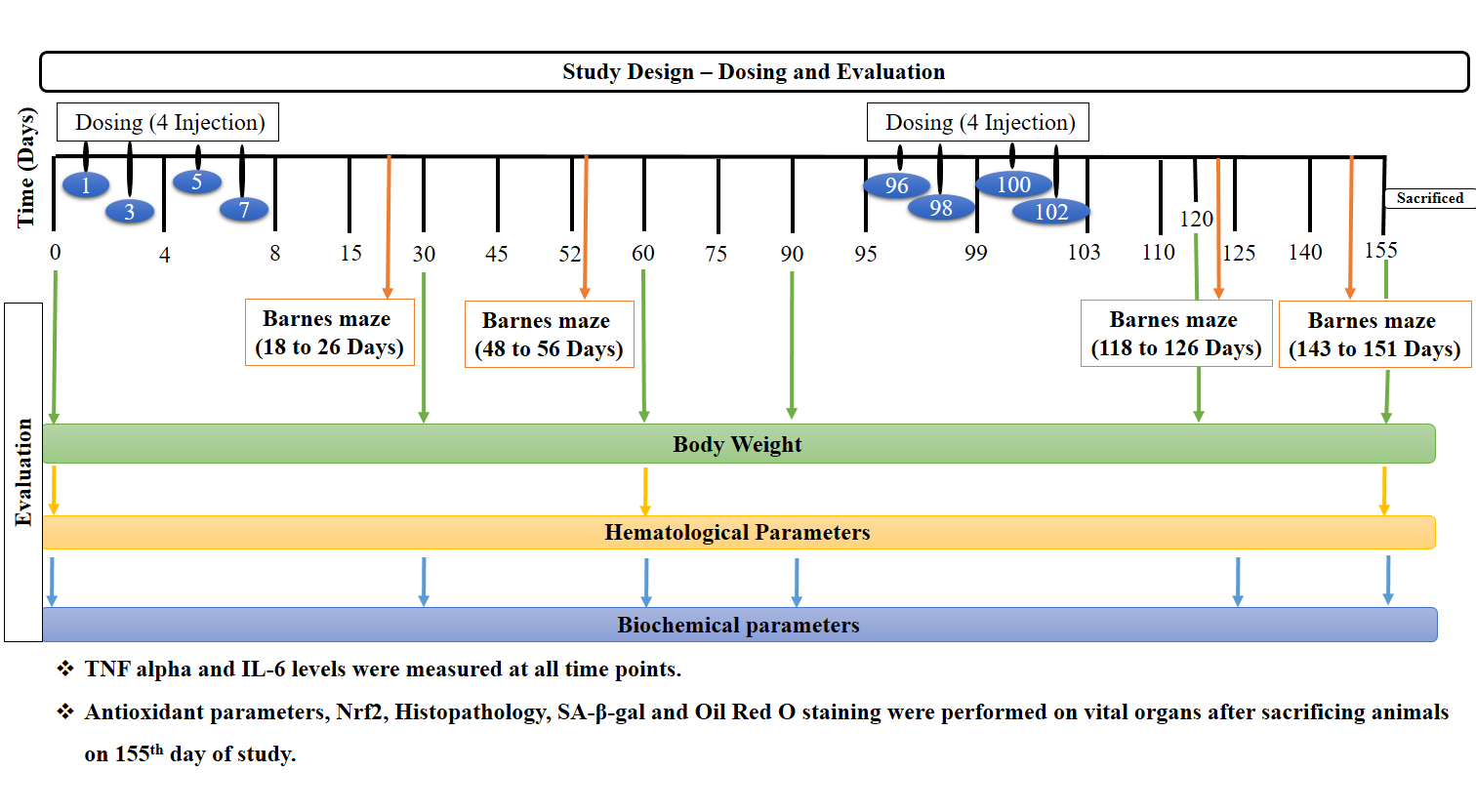
**

**Supplementary Figure 5:** Schematic representation of the study design with timeline indicating plasma fraction treatments as well as cognitive, physical, hematological and biochemical tests.


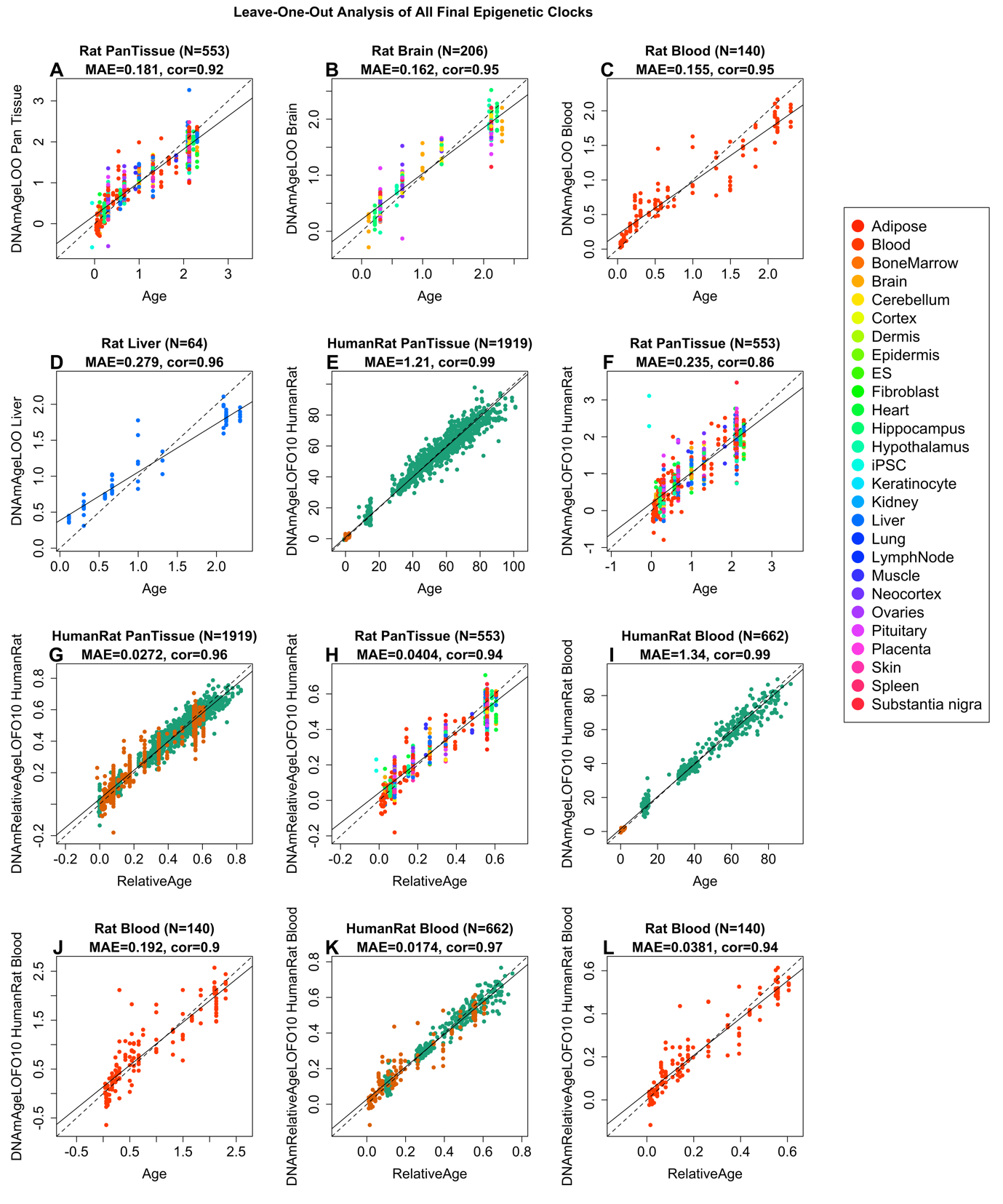


**Supplementary Figure 6:** This figure is analogous to Figure 1, but it reports estimates of the predictive accuracy of the final version of the rat clocks. Cross-validation was carried out on an increased number of rat tissues (n=553) by combining the original training data (n=503) with rat tissues from untreated animals of the second test data set.

**
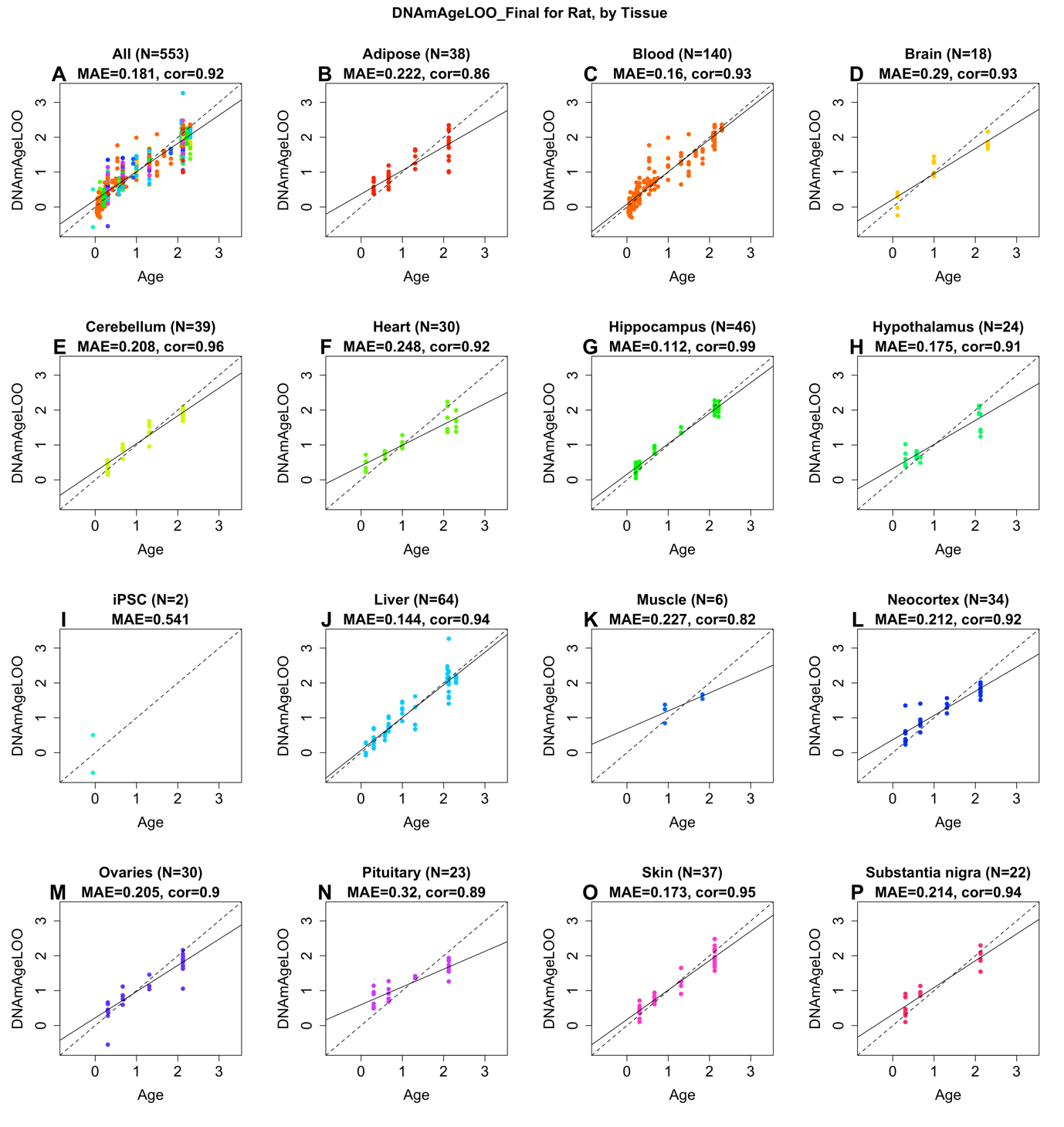
**

**Supplementary Figure 7**: Estimating the accuracy of the final version of the pan tissue rat clock. Each panel corresponds to a different source of DNA in rat tissue. Leave one out cross validation estimates of DNAmAge (y-axis) versus chronological age.


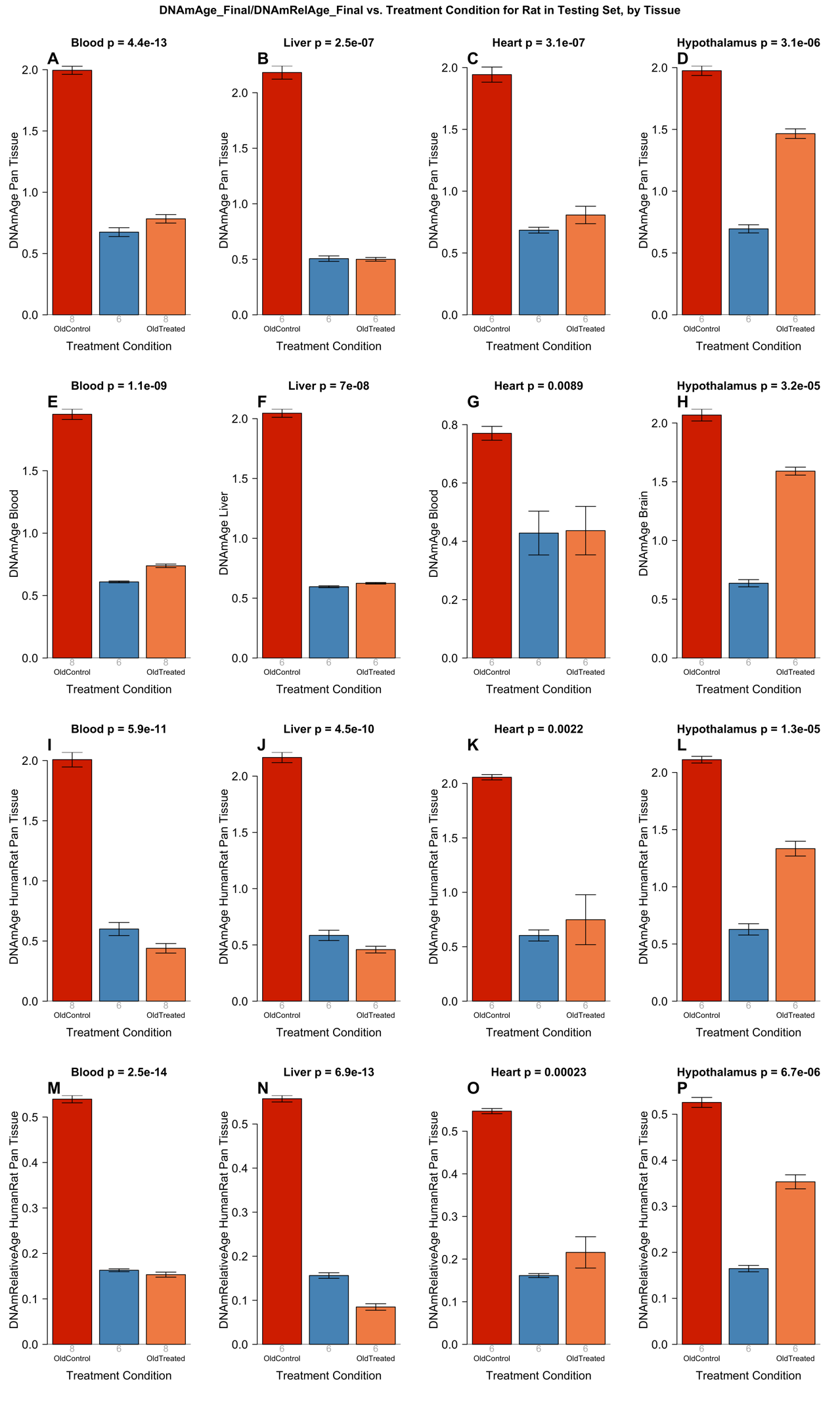


**Supplementary Figure 8**. **Epigenetic clock analysis of plasma fraction treatment based on the final version of epigenetic clocks.** This figure is analogous to Figure 2, but it reports results for the final version of the epigenetic clock based on an increased number of rat tissues (n=567) (resulting from combining the original n=517 original training data with rat tissues from untreated animals of the test data set). Six epigenetic clocks applied to independent test data from four rat tissue type (columns): blood, liver, heart, and hypothalamus. A-D) Rat pan-tissue clock. E) Rat blood clock applied to blood. F) Rat liver clock applied to liver. G) Rat blood clock applied to heart. H) Rat brain clock applied to hypothalamus. I-L) Human-rat clock measure of absolute age. M-P) Human-rat clock measure of relative age defined as age/maximum species lifespan. Each bar-plot reports the mean value and one standard error. Student T-test p values result from a 2-group comparison of old controls (left bar) versus old, treated samples (right bar), i.e. the young controls were omitted.

Blood Liver Heart Hypothalamus

**
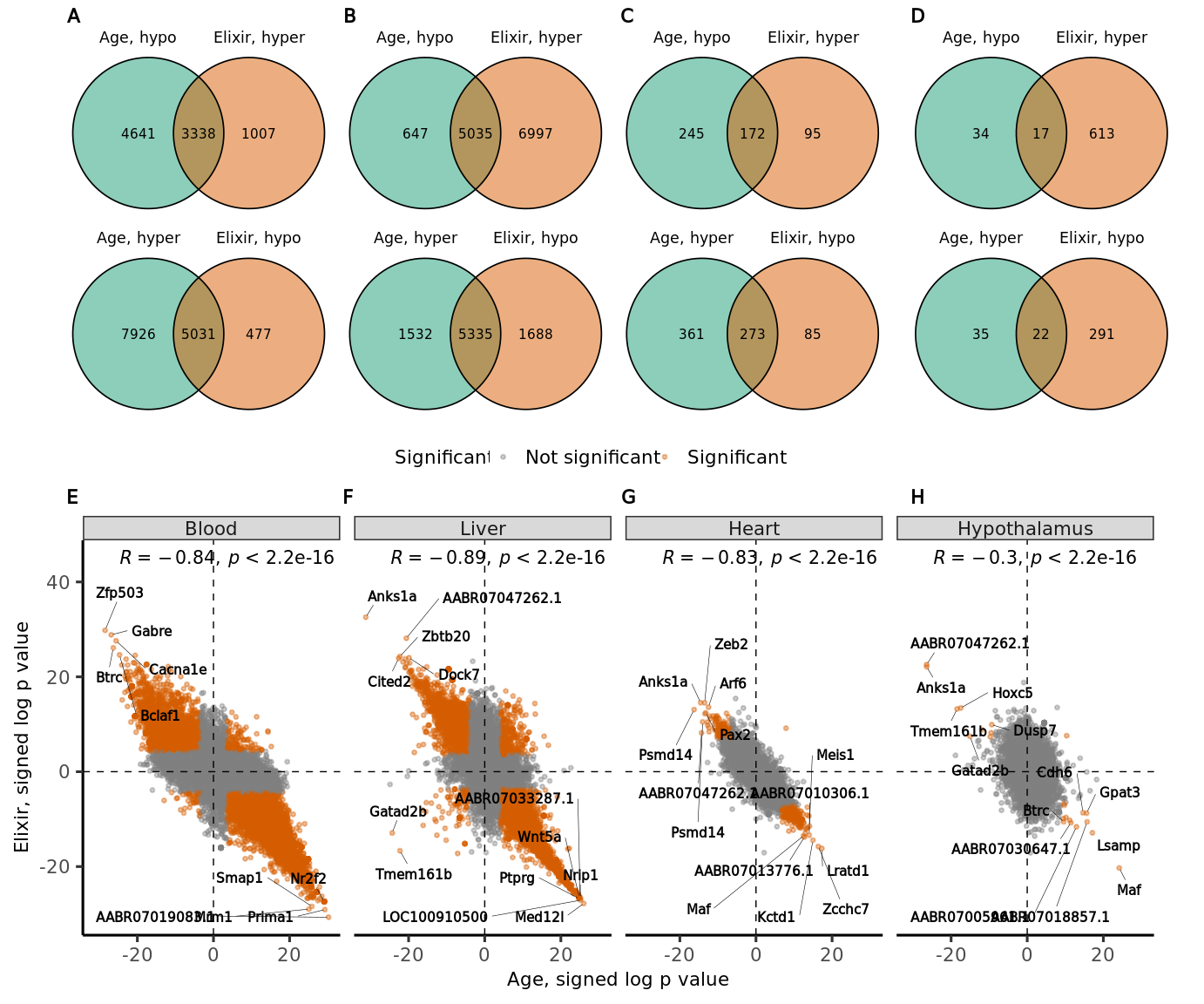
**

**Supplementary Figure 9 (version 1): EWAS results for age versus EWAS results of plasma fraction treatment by tissue type.** This figure portrays two distinct versions of the EWAS study, differentiated by their respective definitions of aging effects.

In Version 1, age effects are determined by comparing old control rats to young ones within the test dataset. This analysis may carry potential bias, as samples from old control animals are employed in assessing both age effects (x-axis) and treatment effects (y-axis). To mitigate this concern, Version 2 of the figure (below) illustrates an alternative approach: age effects are calculated exclusively using independent controls, thus ensuring a more unbiased analysis. The Venn diagrams in version 1 indicate the overlaps of significantly differentially hyper- or hypo-methylated aging and E5 affected CpGs across multiple tissue types: blood (A), liver (B), heart (C), and hypothalamus (D). We used a false discovery rate threshold of 0.05 for both aging and treatment effects (Methods). E-H) Each dot corresponds to a CpG on the mammalian array. Aging effects (x-axis) versus E5 rejuvenation effects (y-axis) in specific rat tissue: blood (E), liver (F), heart (G), and hypothalamus (H). Signed P values were defined as follows: sign(FC) * log(p value). Orange color indicates genes with significant differential methylation in both comparisons. Positive/negative values correspond to gain/loss of methylation associated with the treatment.


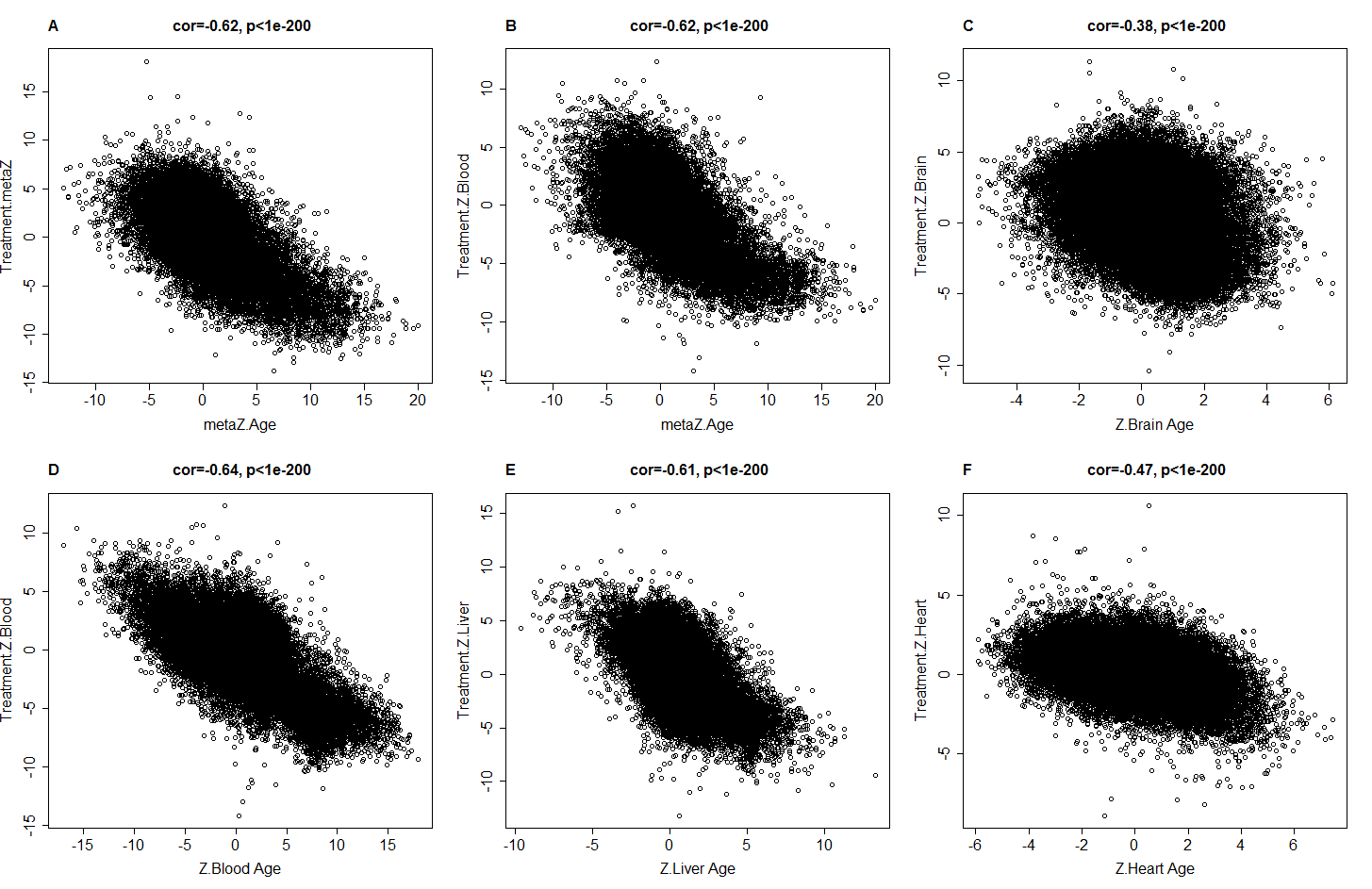


**Supplementary Figure 9 (version 2): EWAS results for age versus EWAS results of plasma fraction treatment.** This version of our EWAS study is less biased than version 1. Each dot corresponds to a CpG on the mammalian array. The x axis reports Z statistics from a correlation test of CpG methylation versus chronological age (R function “standardScreeningNumericTrait” in the WGCNA package). Positive/negative values correspond to positive/negative correlation coefficients with age, respectively. The y-axis reports a Z statistic for the treatment effect. Positive/negative values correspond to gain/loss of methylation associated with the treatment. A-B) The x axis corresponds to a meta-analysis of age effects across all rat tissues (Adipose, Blood, Brain, Cerebellum, Heart, Hippocampus, Hypothalamus, Liver, Neocortex, Ovaries, Pituitary, Skin, Substantia nigra). Age effects in C) Brain, D) Blood, E) Liver, F) Heart. A) Treatment effects across four tissues. Stouffer's meta-analysis Z statistic across hypothalamus, blood, liver, heart. Treatment effects in individual tissues B) Blood, C) Hypothalamus, D) Blood, E) Liver, F) Heart.

**Supplementary Figure 10A. Physical measurements of rats.** Weight of rats measured at regular intervals at indicated times during the 155-day experiment. Each group measurement was from 6 rats. The plotted data points represent average values from 6 rats each, with standard error of the mean.

**Supplementary Figure 10B.** Measurement of grip strength of the indicated rat groups at various times post-treatment. Each group consists of 6 rats. The plotted data points represent average values from 6 rats each, with corresponding 2 standard errors around the mean. In Panel B, for clarity, the results for grip strength are presented without adjustments for individual body weight variations. It's worth noting that the outcomes remained consistent even after accounting for individual body weight, as the treatment did not influence body weight, evident from panel A. Detailed measurements of each parameter are provided in Supplementary Table S5.

**Old Old Treated Young**


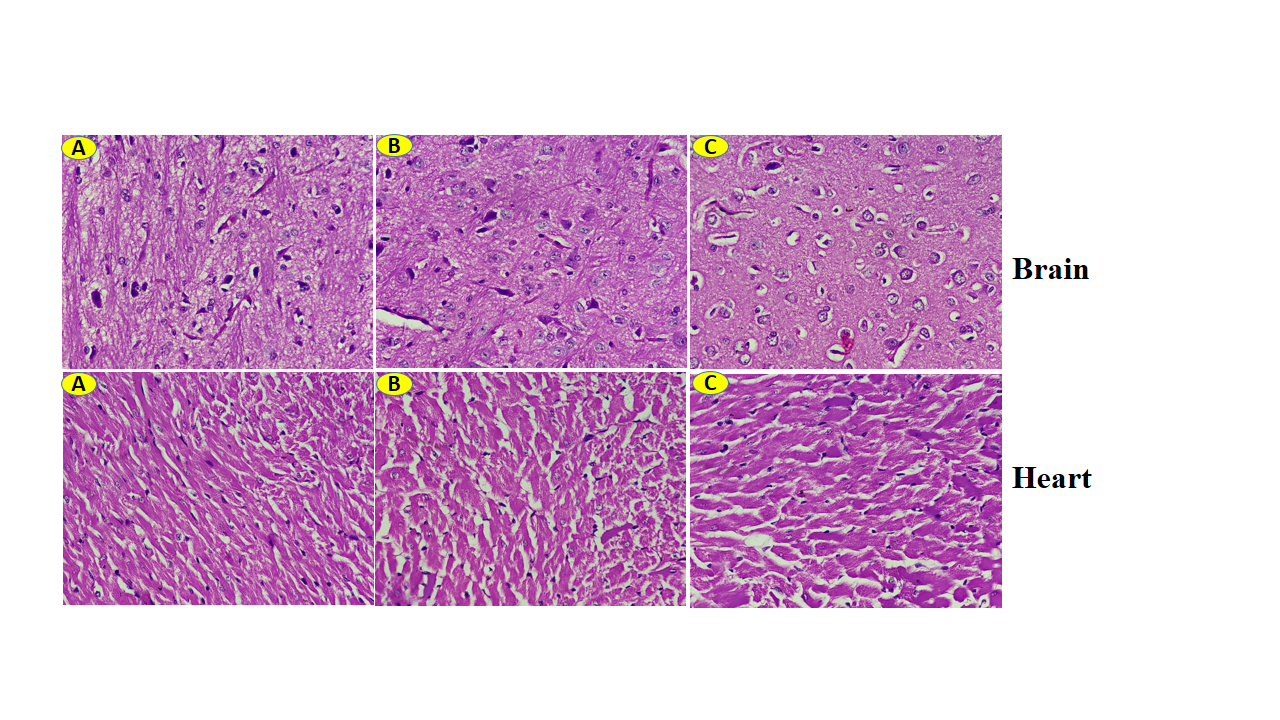


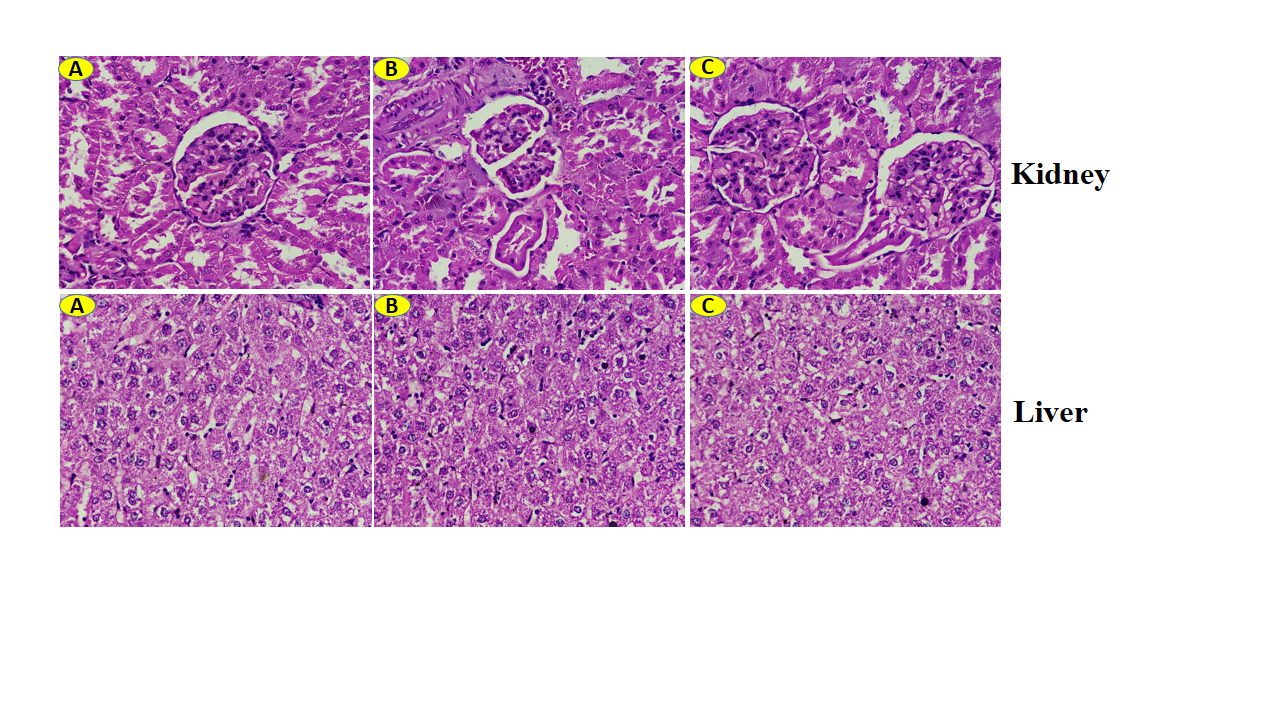


**
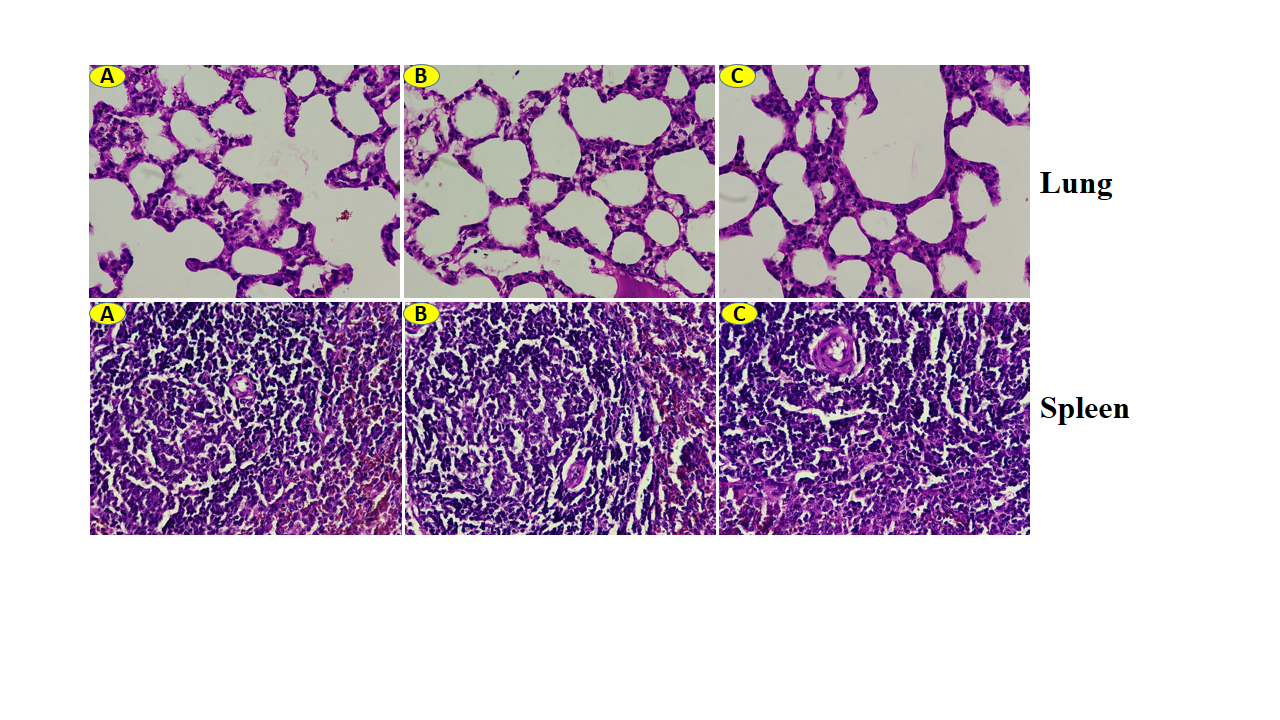
**

**
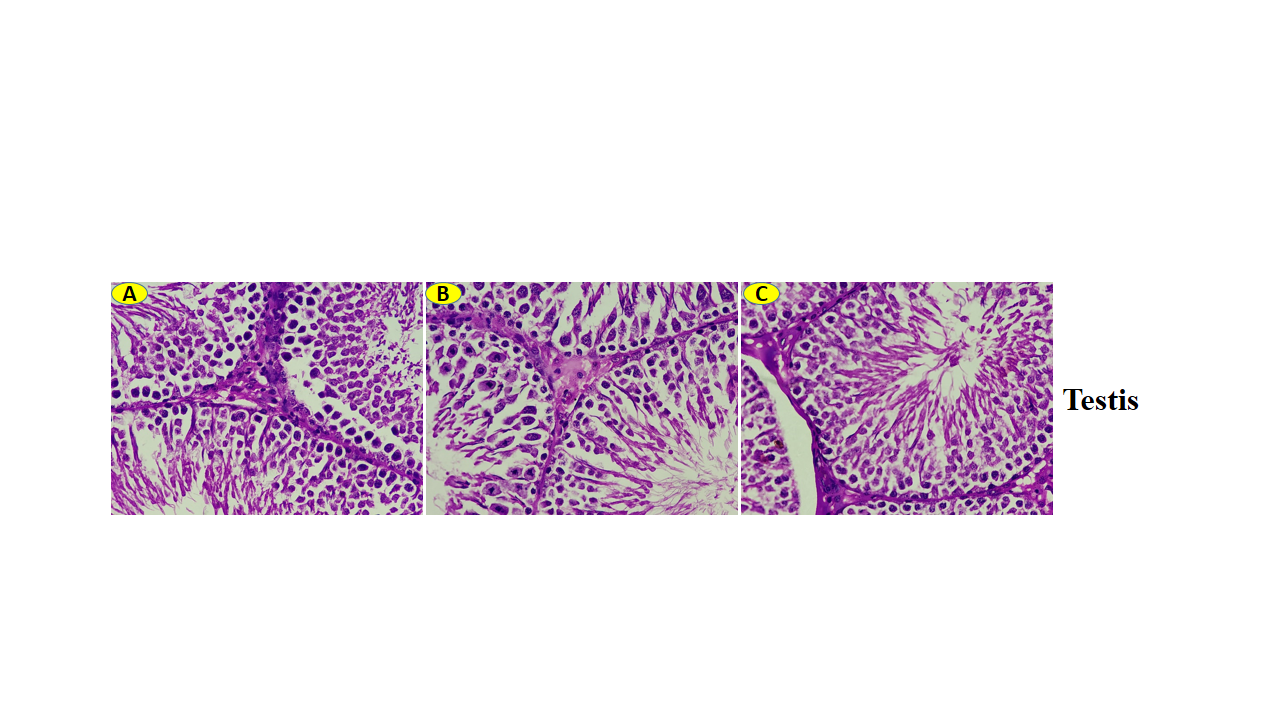
**

**Supplementary Figure 11: Histological analyses of vital organs and tissues of rats employed in experiment**. Images of the left column (A) are from old rats; the right column (C) are images of tissues from young rats while the middle column (B) are images of tissues from old rats treated with plasma fraction. In the liver samples from the old untreated group, we noted minimal, multifocal periportal mononuclear cell infiltration. Such observations were absent in both the treated old and young samples. No abnormalities were observed in the other tissues, regardless of age or treatment. Results of histopathological examination are tabulated in Supplementary Table S6.


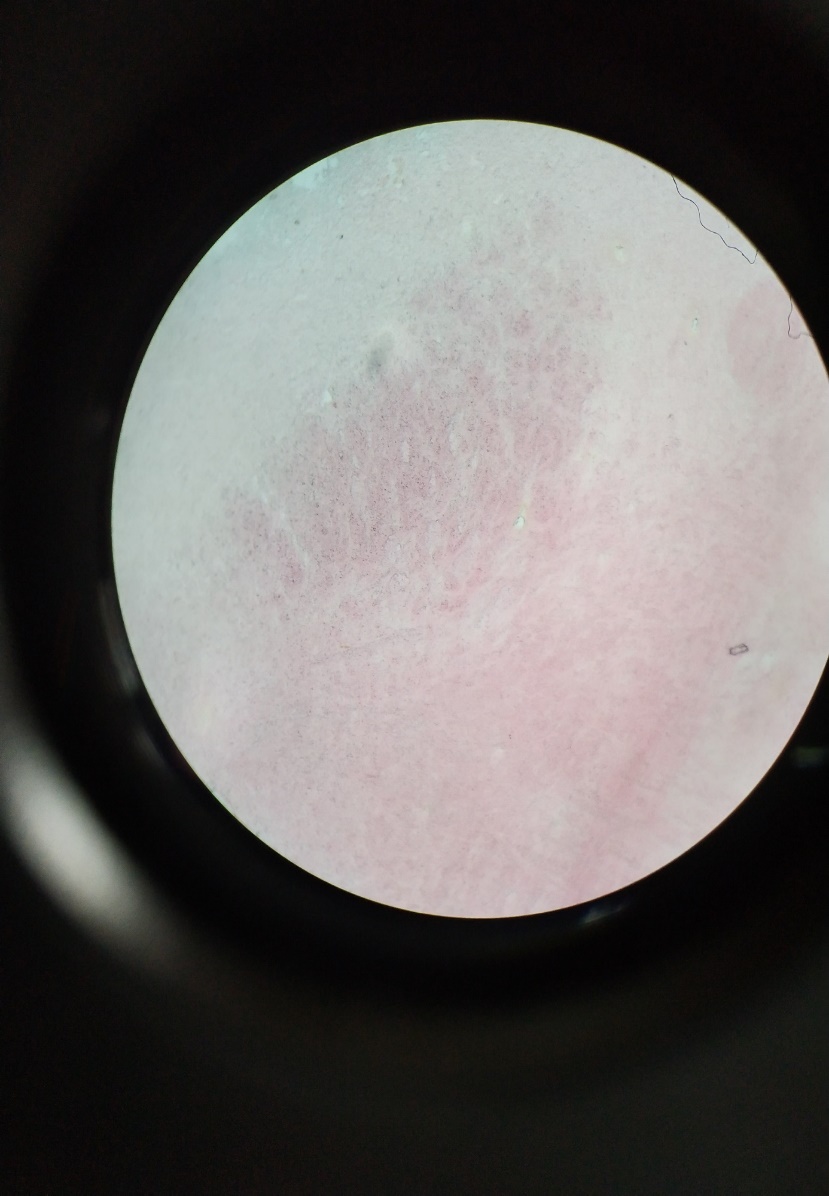

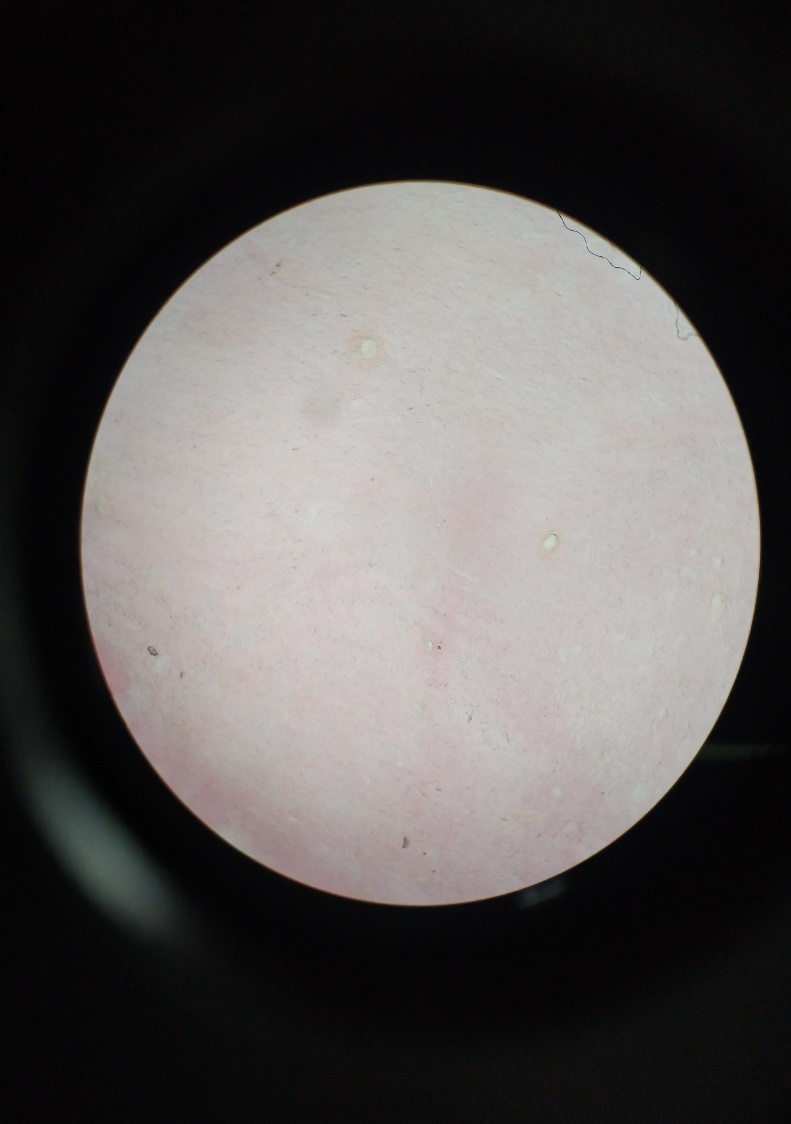

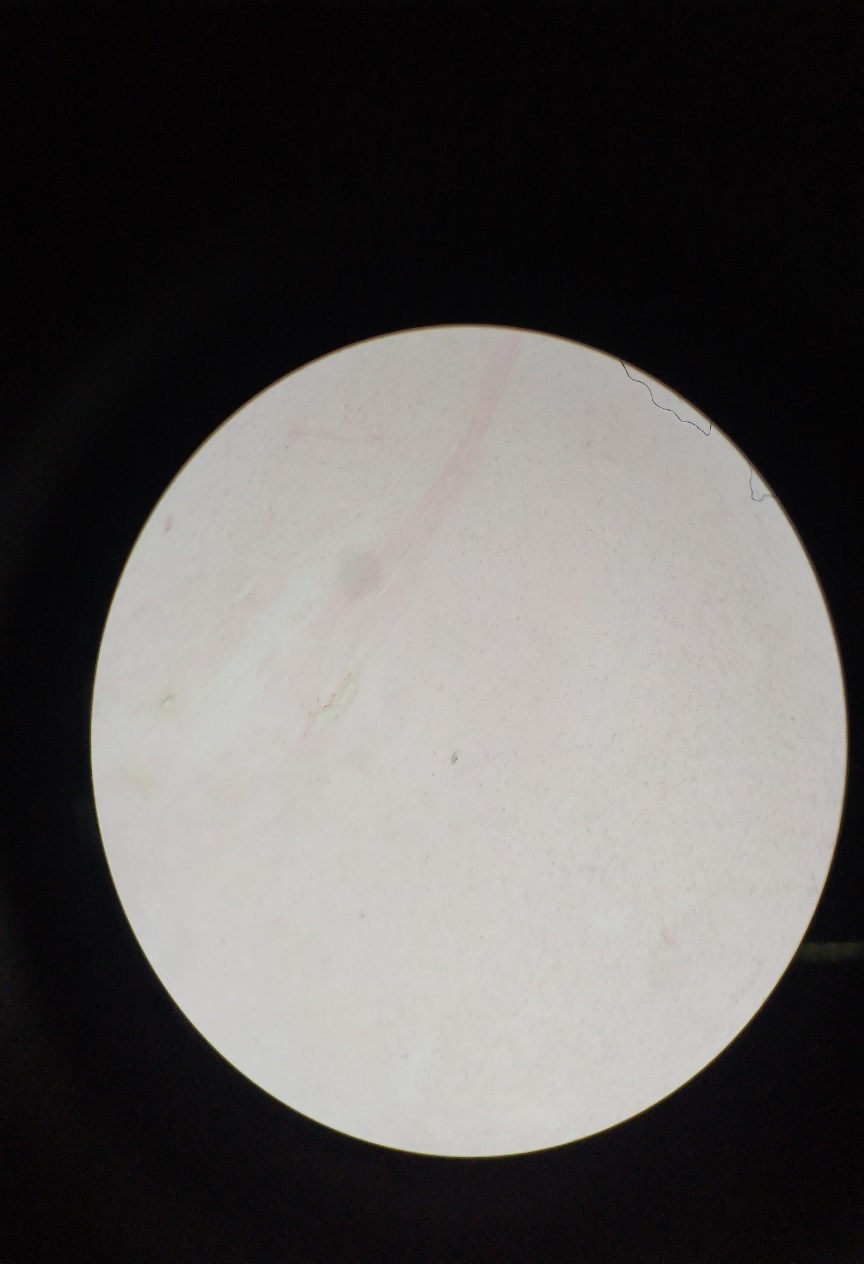

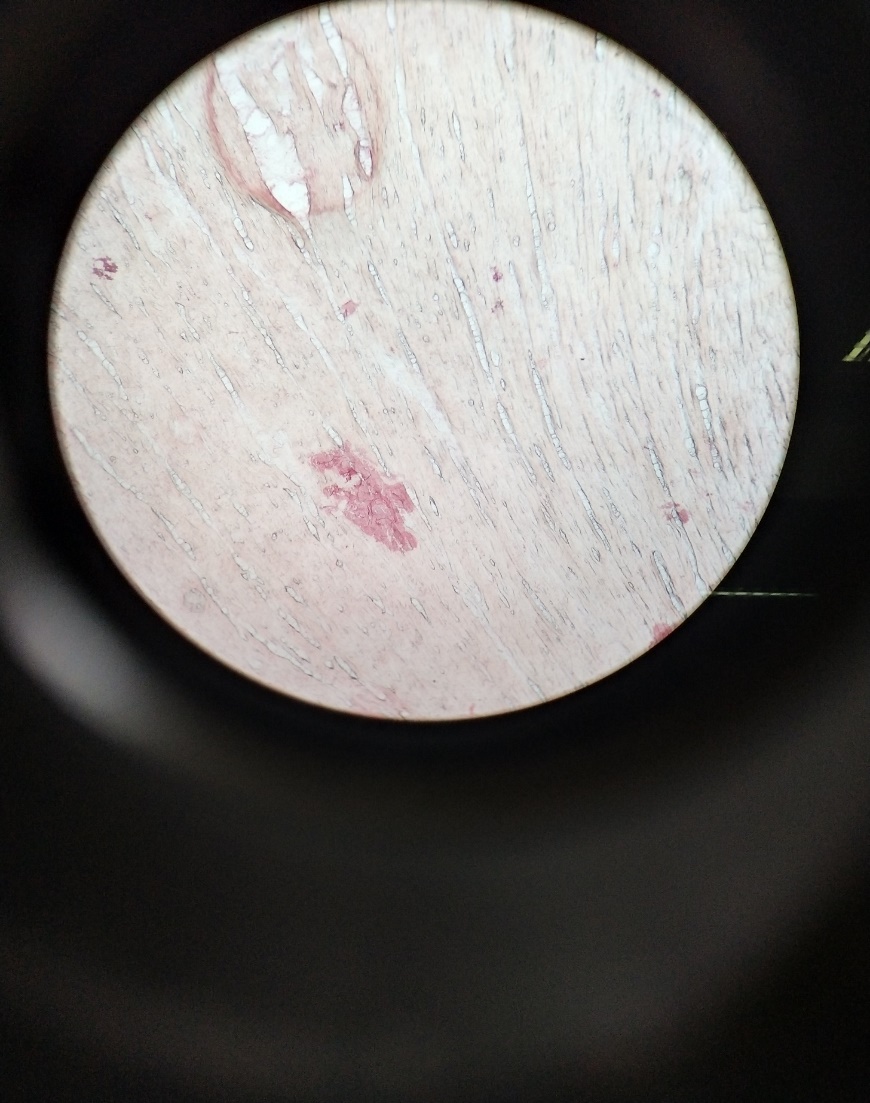

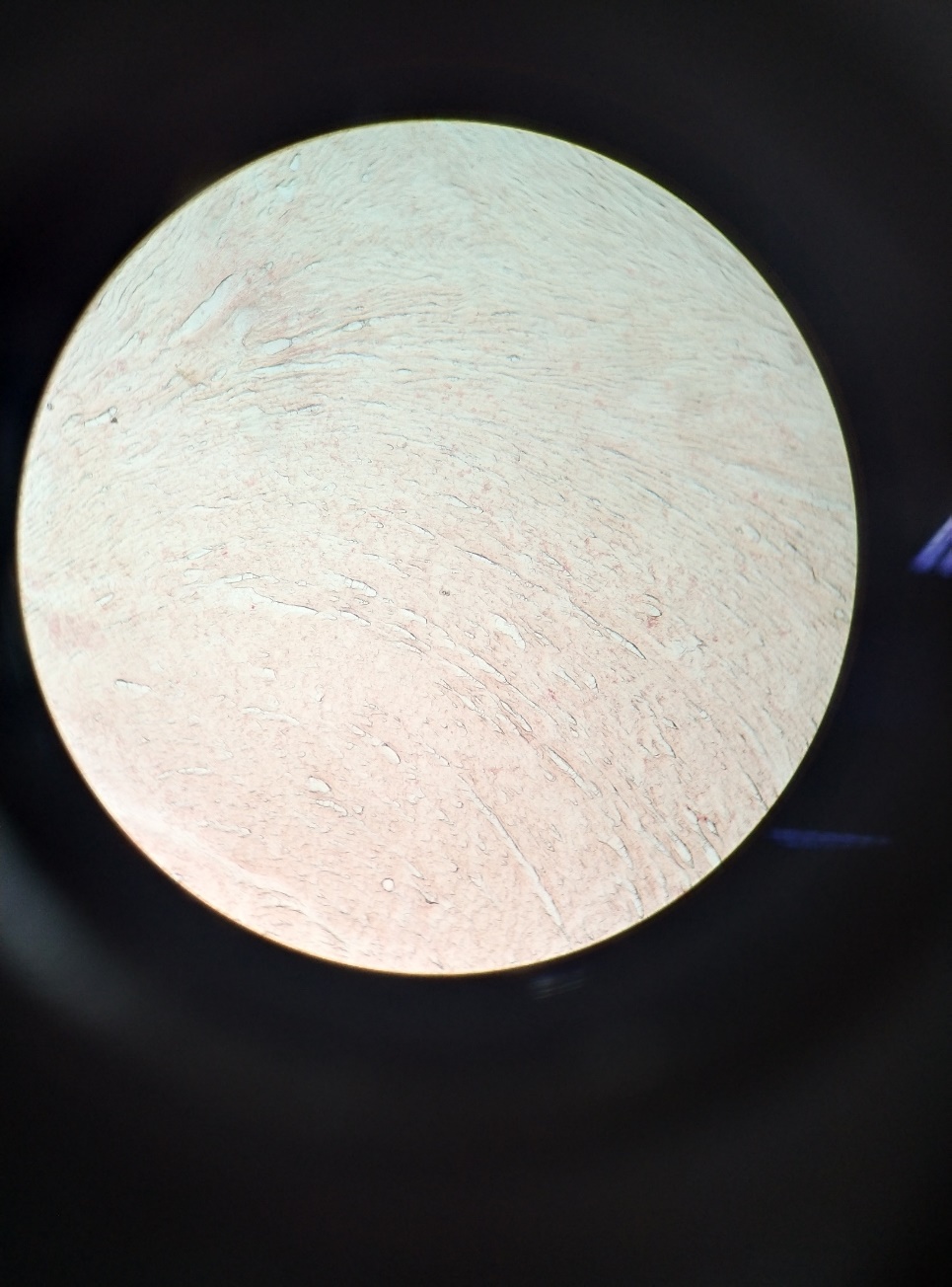

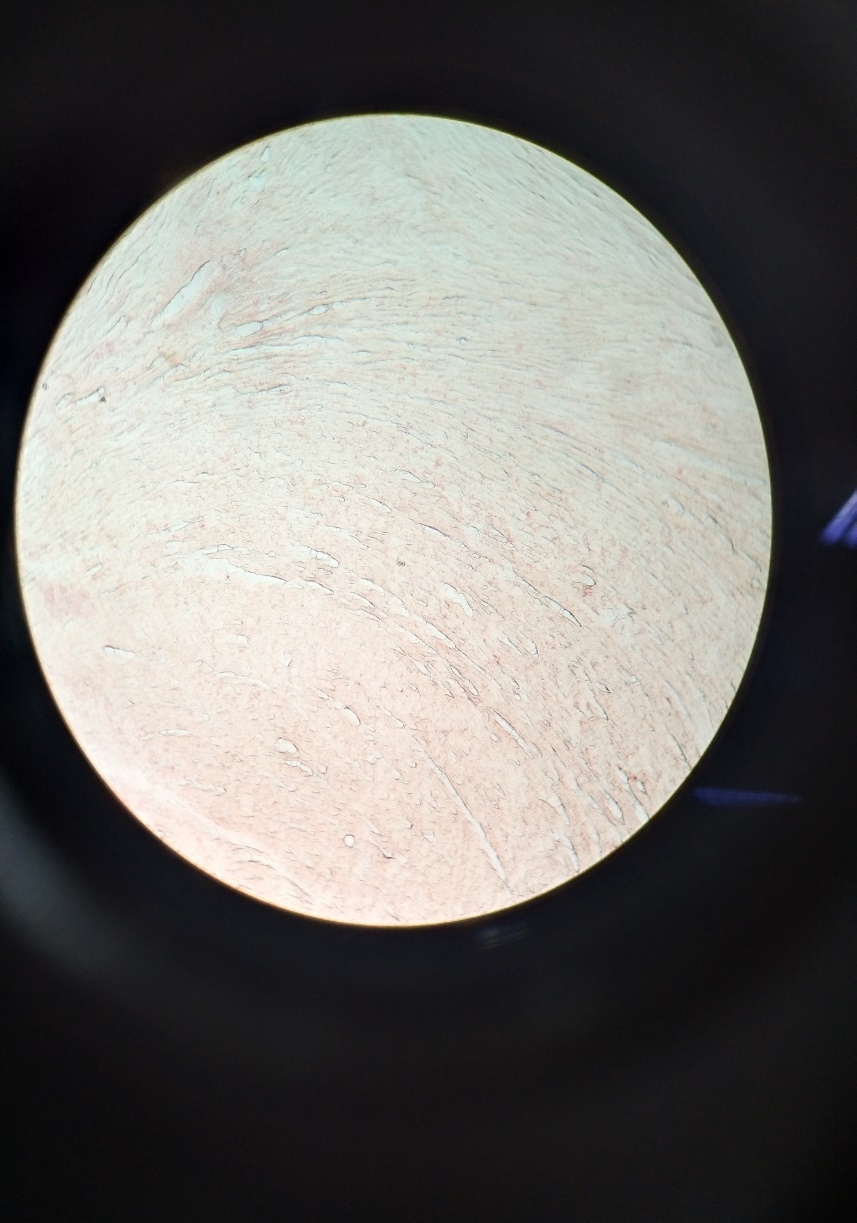

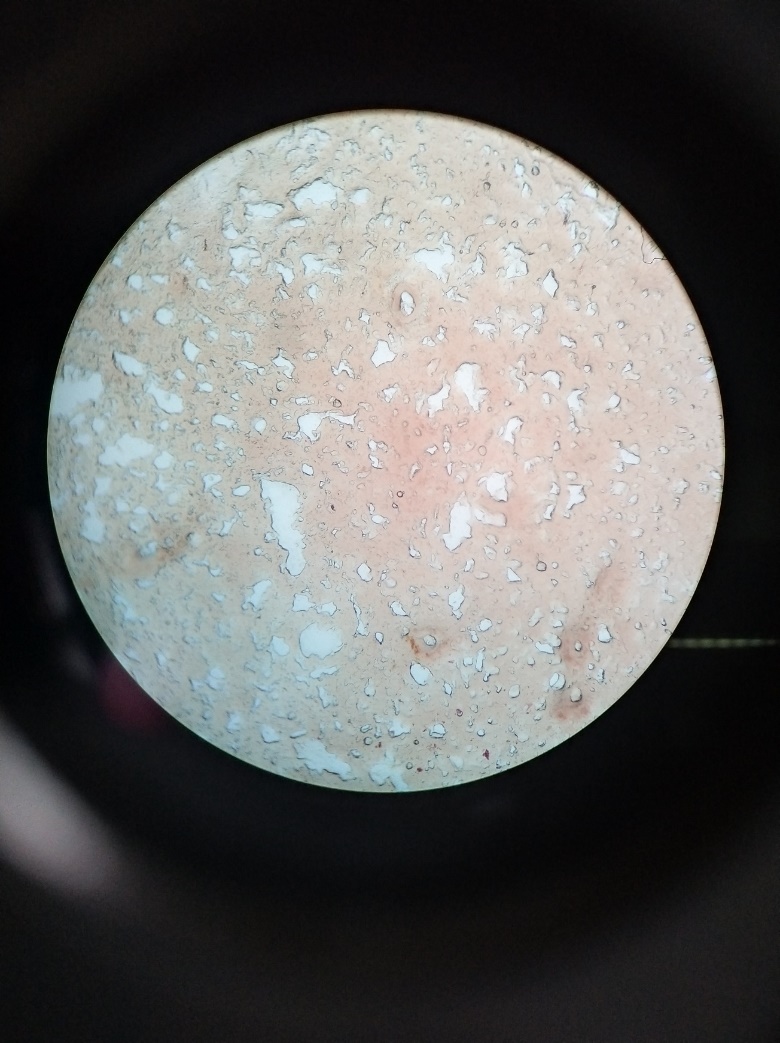

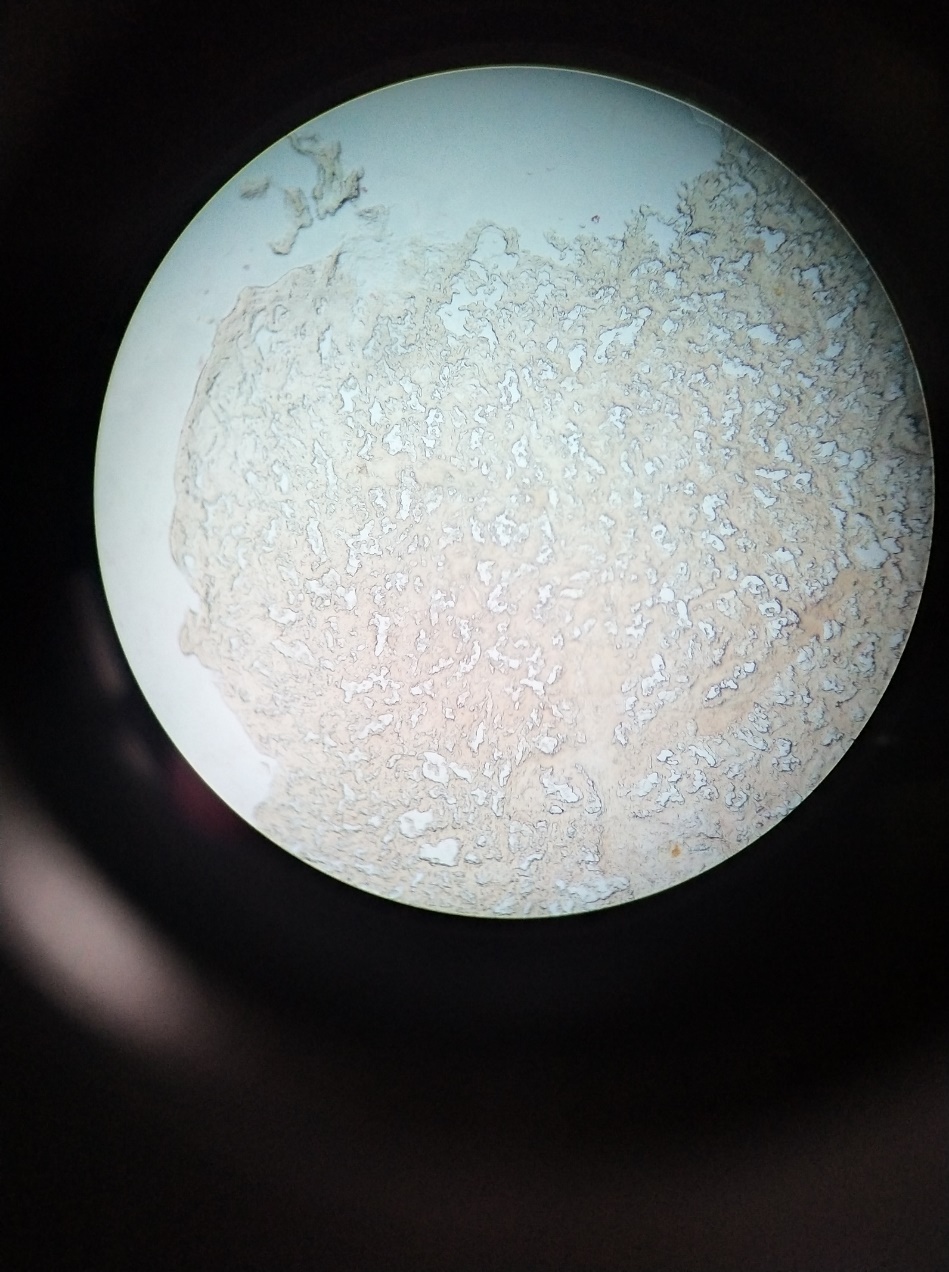

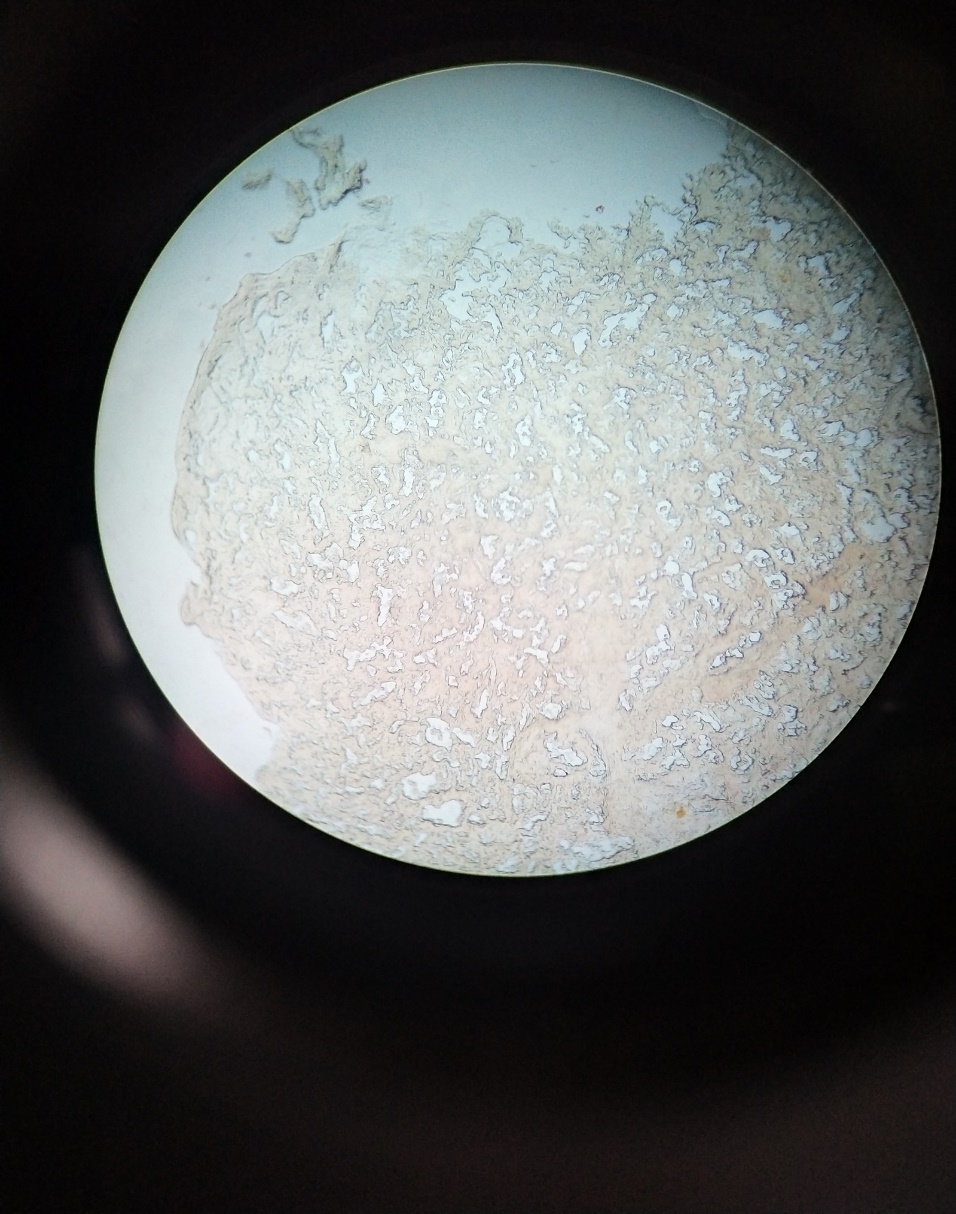

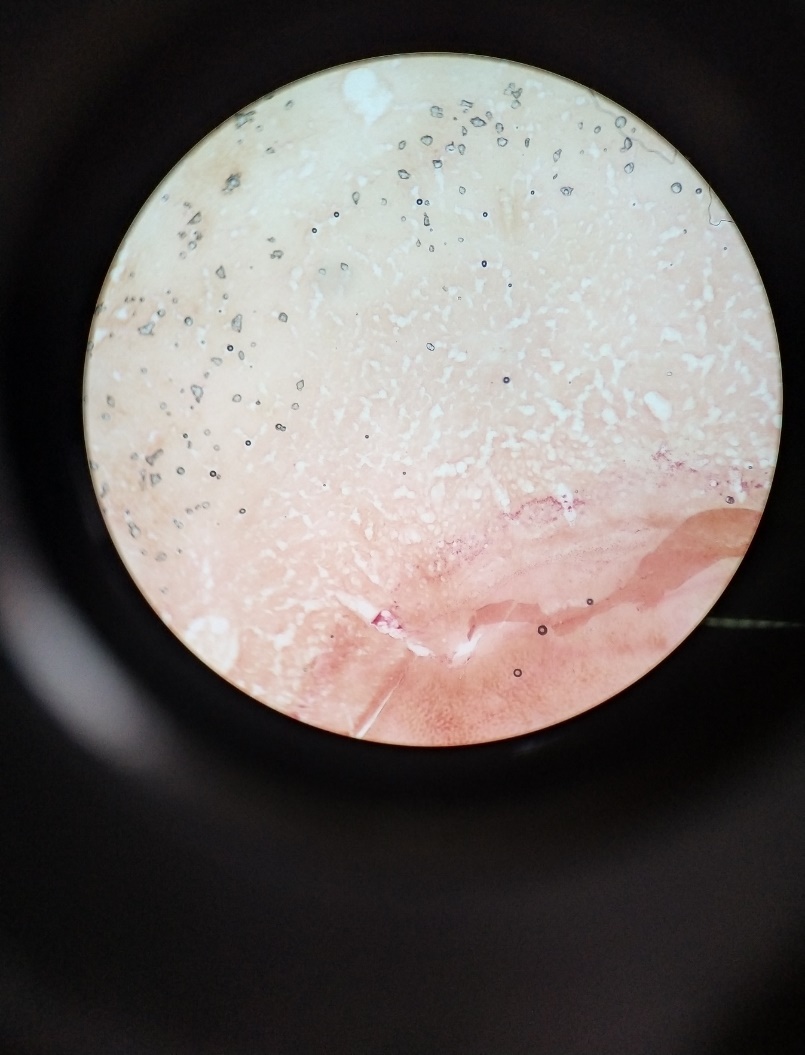

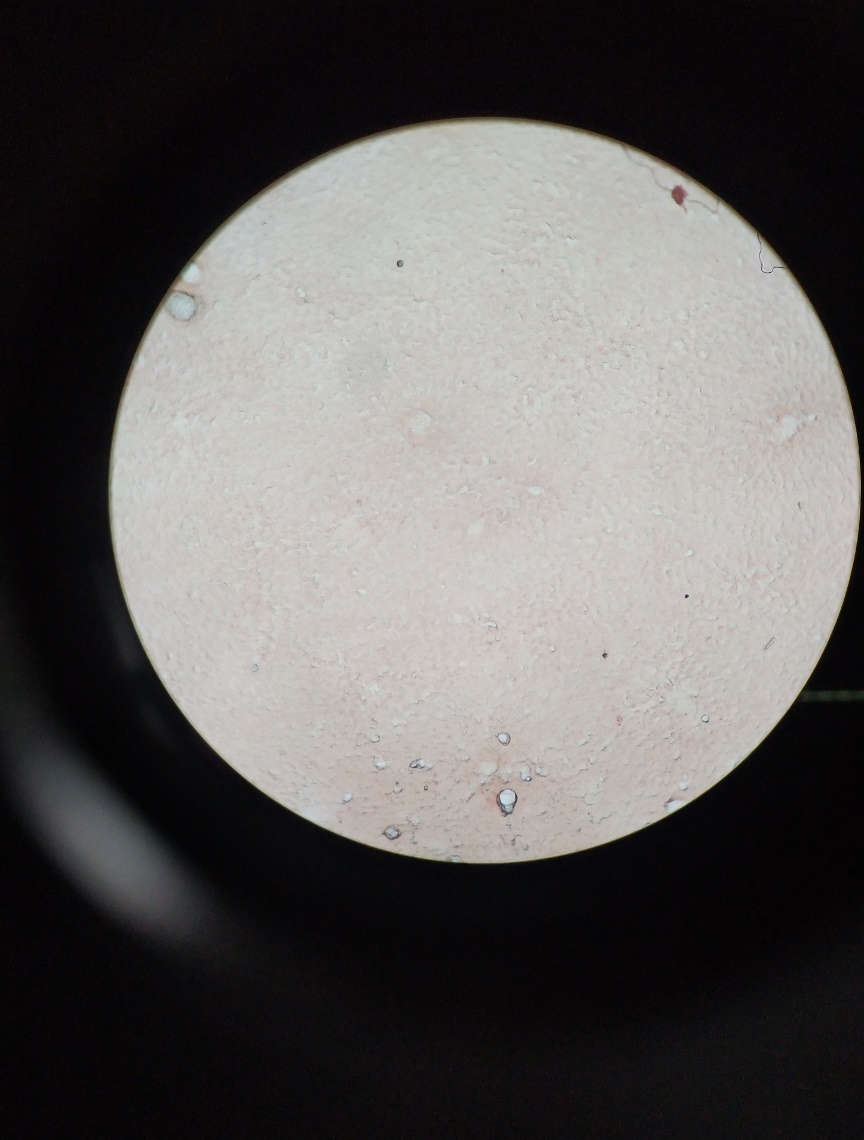

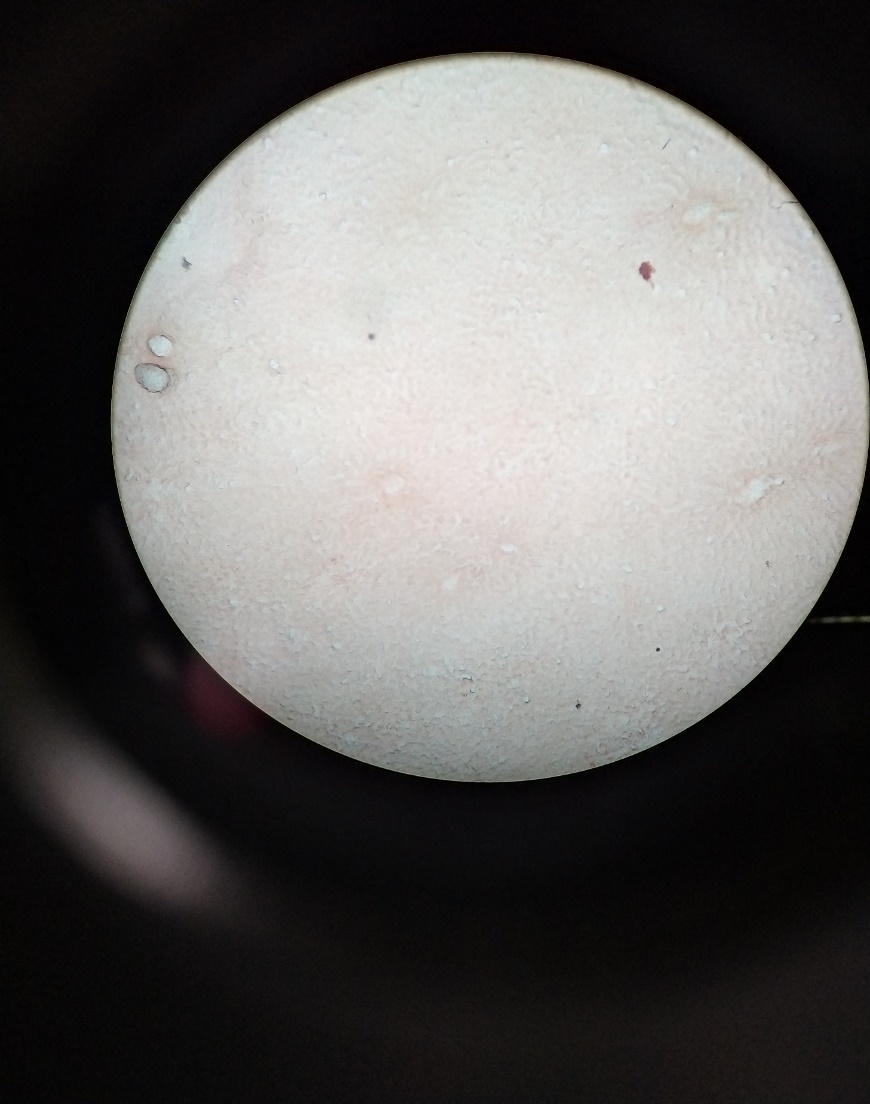


Liver

Old Rat

Treated Old Rat

Young Rat

Lung

Brain

Heart

**Supplementary Figure 12: Oil Red O staining**. Tissues from old untreated rats, Plasma fraction-treated old rats and young rats were subjected to Oil Red O staining to reveal accumulation of fat in tissues.

**Supplementary Figure 13:** **Haematological properties**. Effects of plasma fraction treatment on haematological properties of rats at 0, 60 and 155 days from the start of experiment. Hb = haemoglobin, RBC = red blood cell count, MCV = mean corpuscular volume, MCH = mean corpuscular haemoglobin, MCHC = mean corpuscular haemoglobin concentration, HCT = haematocrit and WBC = white blood cells. Red dots represent data points of old rats, orange dots represent treated old rats and blue represents young rats. The plotted data points represent average values from 6 rats each, with 2 standard errors around the mean. Detailed measurements of each parameter are provided in Supplementary Table S7.

|  |  |
| --- | --- |
|  |  |
|  |  |

**Supplementary Figure 14. Evaluating the effect of E5 in both male and female Sprague Dawley rats**. Upper panels: TNA alpha measurements (y-axis). Middle panels: IL-6 levels in picogram/ml. Lower panels: Grip strength assessment. The left panels present data for male rats, while the right panels detail results for female rats. The units for pro-inflammatory cytokine levels determined using a sandwich ELISA (Enzyme-Linked Immunosorbent Assay) kit are expressed in picograms per milliliter. Details can be found in Rat Tumor Necrosis Factor A (TNF-a) ELISA kit from KinesisDx, catalogue number: K11-0765. The grip strength in rats was measured using a grip strength meter, which records the peak pull force exerted by the rat in units of "grams" (g). At baseline, the rats were 26 months old. The study analyzed 12 male and 12 female rats. Half of the animals were treated with E5. The controls were treated with saline.

**Supplementary Figure 15. Epigenetic clock analysis in blood of male and female rats**. This is an independent validation study of epigenetic clocks that is based on a different set of Sprague Dawley rats of both sexes. A) Final version of the rat clock for blood. Baseline measurement (x-axis) versus follow up measurement (15 days after treatment, y-axis). Points (rats) are colored by treatment: red=treated by E5, black=treated with saline only. B,D,E) Difference between follow up measurement and baseline measurement (y-axis) versus treatment status in B) all rats, D) female rats only, E) male rats only. C) is analogous to B) but uses the pan tissue clock for rats.

Panels in the second row (F,G,H,I,J) are analogous to those in the first row but the analysis omitted one control rat (corresponding to the black dot in the lower right of panel A).

The title of the bar plots reports the results of a non-parametric group comparison test (unadjusted two-sided p value from the Kruskal Wallis test). The rotated grey number underneath each bar reports the group size. Each bar plot reports the mean value and one standard error.

**Supplementary Figure 16. Gel Electrophoresis of Extracted RNAs from E5 Sample Visualized on GelDock.** A molecular ladder of 1,000 base pairs was used as a reference. Two distinct bands were evident in the E5 sample: one at 100 base pairs and a more concentrated band above 1,000 base pairs. The presence of RNA masses exceeding 1,000 base pairs, as noted in literature, indicates the potential presence of long chain noncoding RNAs (lncRNAs). This suggests a predominant presence of lncRNAs in the E5 samples.

Information regarding the RNA procedure and gel electrophoresis is provided in the Supplementary Methods section below.


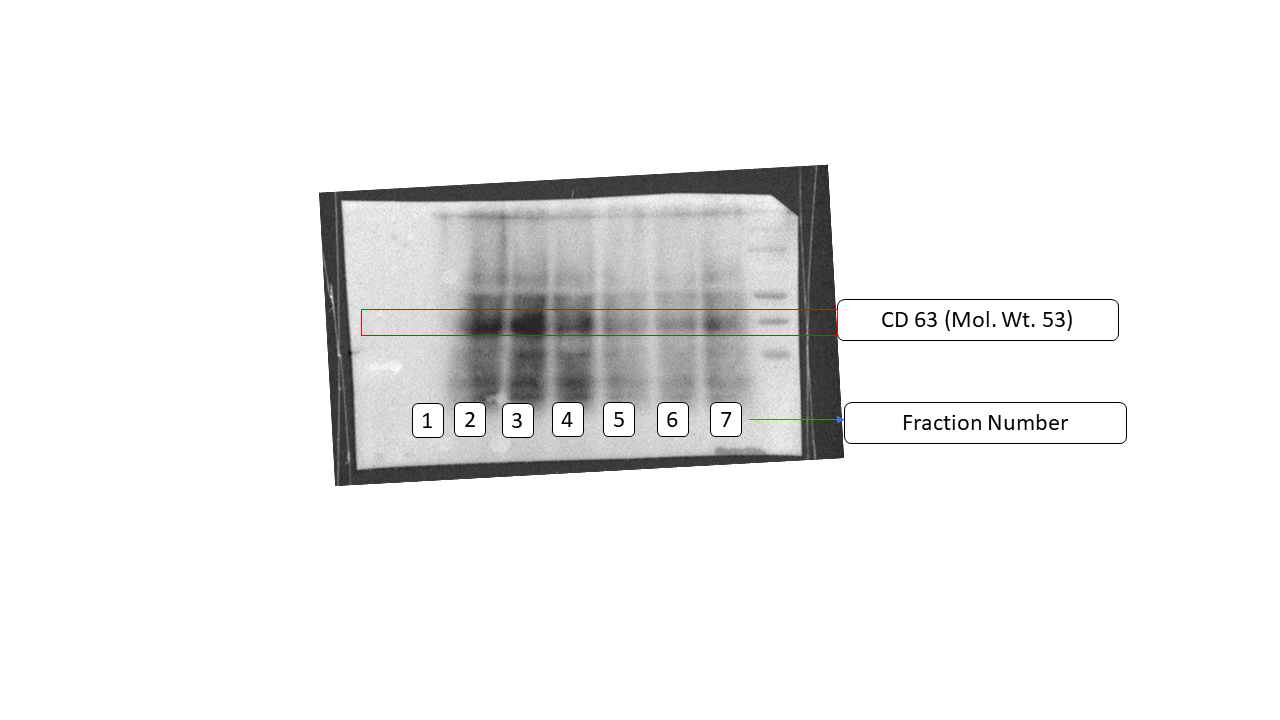

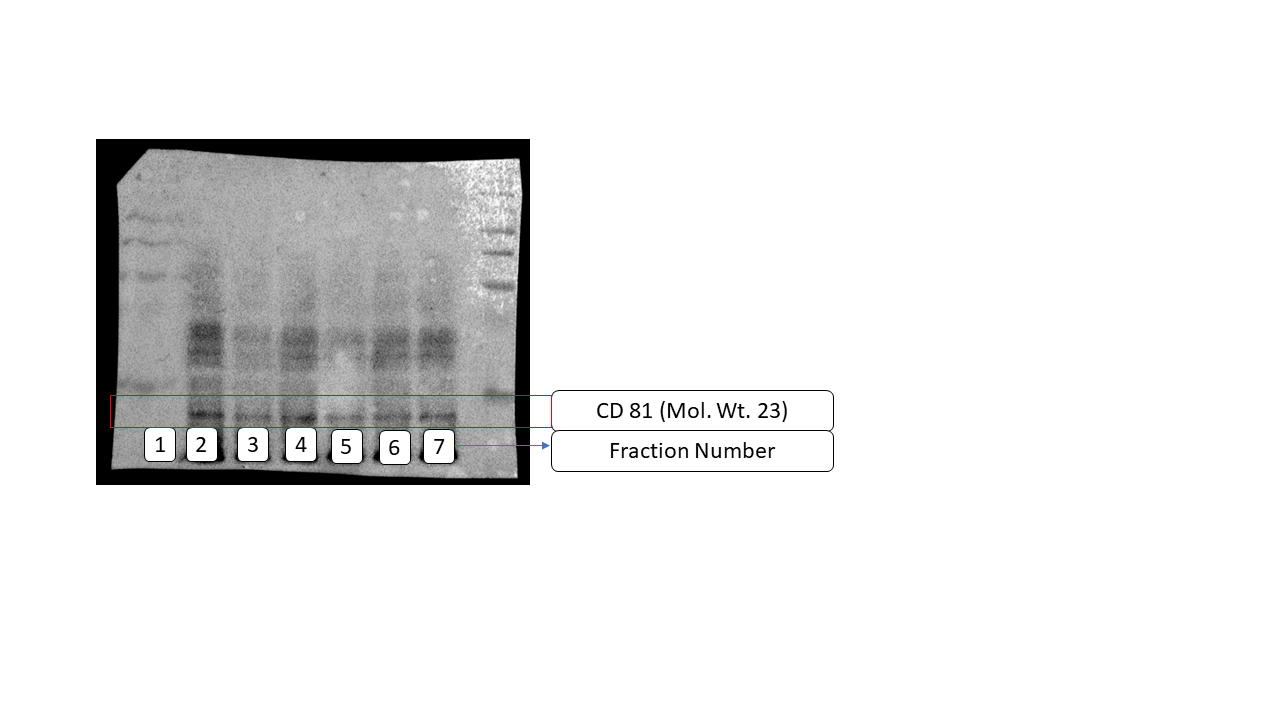


**Supplementary Figure 17. Western Blot Study showing the fractions of E5 contain proteins CD63 and CD81**. Expression of CD63 and CD81 in E5 Fractions was determined using CD63 (1:1000) and CD81 (Rabbit 1:1000) (ABclonal Technology, USA) primary antibody and peroxidase-labeled goat anti-rabbit IgG secondary antibody. Additional details can be found in supplementary methods.

**Supplementary Tables**

| **Tissue** | **N** | **No. Female** | **Mean Age** | **Min. Age** | **Max. Age** |
| --- | --- | --- | --- | --- | --- |
| Adipose | 38 | 38 | 1.2 | 0.305 | 2.12 |
| Blood | 126 | 72 | 0.93 | 0.0384 | 2.3 |
| Whole Brain | 18 | 3 | 1.3 | 0.115 | 2.3 |
| Cerebellum | 39 | 39 | 1.2 | 0.305 | 2.12 |
| Heart | 18 | 3 | 1.3 | 0.115 | 2.3 |
| Hippocampus | 46 | 46 | 1.1 | 0.216 | 2.22 |
| Hypothalamus | 12 | 22 | 1.2 | 0.305 | 2.12 |
| iPSC | 2 | NA | 0 | 0 | 0 |
| Muscle | 6 |  | 1.5 | 1.0 | 2.0 |
| Liver | 52 | 37 | 1.2 | 0.115 | 2.3 |
| Neocortex | 34 | 34 | 1.2 | 0.305 | 2.12 |
| Ovaries | 30 | 30 | 1.2 | 0.305 | 2.12 |
| Pituitary | 23 | 23 | 1.2 | 0.305 | 2.12 |
| Skin | 37 | 37 | 1.2 | 0.305 | 2.12 |
| Substantia nigra | 22 | 22 | 1.1 | 0.305 | 2.12 |

**Supplementary Table S1.** Description of biological materials for training the preliminary epigenetic clocks for rats (N=503). Other supplementary tables provide details on the test data. N=Total number of tissues. Number of females. Age: mean, minimum and maximum in units of years.

| **Sr. No** | **Age in Weeks** | **Age in Months** | **Number of Animal gender-wise (n=6)** | **Blood sample** | **Tissue samples** | **Total Samples** |
| --- | --- | --- | --- | --- | --- | --- |
| 1 | 3 | 0.69 | 3- Male and 3- Female | 6 | - | 6 |
| 2 | 6 | 1.38 | 3- Male and 3- Female | 6 | Heart, Liver, Brain (18 samples) | 24 |
| 3 | 12 | 2.76 | 3- Male and 3- Female | 6 | - | 6 |
| 4 | 28 | 6.44 | 6- Male | 6 | - | 6 |
| 5 | 52 | 12.0 | 6- Male | 6 | Heart, Liver, Brain (18 samples) | 24 |
| 6 | 78 | 18.0 | 3- Male and 3- Female | 6 |  | 6 |
| 7 | 120 | 27.6 | 6- Male | 6 | Heart, Liver, Brain (18 samples) | 24 |
| **Total samples (42+54=96)** | | | | **42** | **54** | **96** |

**Supplementary Table S2.** Description of the Sprague Dawley rat samples provided by Dr Kavita Singh for training of rat clock.

| **Experiment** | **Group** | **Age in Weeks** | **Age in Months** | **Number of Animal (n=6)** | **Blood sample** | **Tissue samples** | **Total Samples** |
| --- | --- | --- | --- | --- | --- | --- | --- |
| 1 | Old Control | 109 | 25.0 | 6 Male | 6+2 replicates | Heart, Liver, Hypothalamus (6 samples each) | 26 |
| 1 | Old Treatment | 109 | 25.0 | 6 Male | 6+2 replicates | Heart, Liver, Hypothalamus (6 samples each) | 26 |
| 1 | Young Control | 30 | 6.9 | 6 Male | 6 | Heart, Liver, Hypothalamus (6 samples each) | 24 |
|  | **Total samples (22+54=76)** | | | | **22** | **54** | **76** |

| **Experiment** | **Group** | **Age in Weeks** | **Age in Months** | **Number of Animal** | **Blood sample** | **Samples** | **Total Samples** |
| --- | --- | --- | --- | --- | --- | --- | --- |
| 2 | Old Control | 113.4 | 26.0 | 9 (5F, 4M) | 2 per animal | Blood draws separated by 2 weeks | 18 |
| 2 | Old Treated | 113.4 | 26.0 | 8 (6F,2M) | 2 per animal | Blood draws separated by 2 weeks | 16 |
|  | **Total samples** | | | |  |  | **34** |

**Supplementary Table S3.** Description of the Sprague Dawley rats employed for testing the effects of the E5 plasma fraction treatment. The two set of independent experiments involved different sets of animals. Experiment 1 profiled multiple organs and tissues from male rats. Experiment 2 analyzed blood methylation data from both male and female rats. Experiment 2 was a longitudinal study based on two blood draws: baseline (before any treatment) and 2 weeks post treatment (E5 treatment or saline control).

| **Figure** | **Tissue** | **Clock** | **Old Ctrl** | **Young Ctrl** | **Old Treated** | **Perc. Rejuv.** | **Ave. Rejuv. By**  **Tissue** |
| --- | --- | --- | --- | --- | --- | --- | --- |
| Fig2B | Liver | DNAmAge Pan Tissue | 2.1322 | 0.5260 | 0.5715 | 73.20% |  |
| Fig2F | Liver | DNAmAge Liver | 2.4875 | 0.7220 | 0.7809 | 68.61% |  |
| Fig2J | Liver | DNAmAge HumanRat Pan Tissue | 4.2965 | 1.0316 | 0.9394 | 78.14% |  |
| Fig2N | Liver | DNAmRelativeAge HumanRat Pan Tissue | 0.6752 | 0.2149 | 0.1444 | 78.62% | 74.64% |
| SuppFig12B | Liver | DNAmAge Pan Tissue | 2.1818 | 0.5057 | 0.4997 | 77.10% |  |
| SuppFig12F | Liver | DNAmAge Liver | 2.0457 | 0.5955 | 0.6243 | 69.48% |  |
| SuppFig12J | Liver | DNAmAge HumanRat Pan Tissue | 2.1661 | 0.5847 | 0.4583 | 78.84% |  |
| SuppFig12N | Liver | DNAmRelativeAge HumanRat Pan Tissue | 0.5575 | 0.1562 | 0.0848 | 84.79% | 77.55% |
| Fig2D | Hypothalamus | DNAmAge Pan Tissue | 1.6158 | 0.6597 | 1.2900 | 20.16% |  |
| Fig2H | Hypothalamus | DNAmAge Brain | 2.3387 | 0.9713 | 1.9615 | 16.13% |  |
| Fig2L | Hypothalamus | DNAmAge HumanRat Pan Tissue | 2.1391 | 0.7041 | 1.4740 | 31.09% |  |
| Fig2P | Hypothalamus | DNAmRelativeAge HumanRat Pan Tissue | 0.4861 | 0.1879 | 0.3392 | 30.22% | 24.40% |
| SuppFig12D | Hypothalamus | DNAmAge Pan Tissue | 1.9749 | 0.6954 | 1.4656 | 25.79% |  |
| SuppFig12H | Hypothalamus | DNAmAge Brain | 2.0678 | 0.6363 | 1.5903 | 23.09% |  |
| SuppFig12L | Hypothalamus | DNAmAge HumanRat Pan Tissue | 2.1123 | 0.6277 | 1.3340 | 36.84% |  |
| SuppFig12P | Hypothalamus | DNAmRelativeAge HumanRat Pan Tissue | 0.5256 | 0.1646 | 0.3532 | 32.81% | 29.64% |
| Fig2C | Heart | DNAmAge Pan Tissue | 1.6065 | 0.8285 | 0.8086 | 49.66% |  |
| Fig2G | Heart | DNAmAge Blood | 0.7898 | 0.4014 | 0.4133 | 47.67% |  |
| Fig2K | Heart | DNAmAge HumanRat Pan Tissue | 3.8106 | 1.7115 | 1.9973 | 47.59% |  |
| Fig2O | Heart | DNAmRelativeAge HumanRat Pan Tissue | 0.5783 | 0.2598 | 0.3409 | 41.06% | 46.49% |
| SuppFig12C | Heart | DNAmAge Pan Tissue | 1.9428 | 0.6844 | 0.8074 | 58.44% |  |
| SuppFig12G | Heart | DNAmAge Blood | 0.7704 | 0.4283 | 0.4367 | 43.32% |  |
| SuppFig12K | Heart | DNAmAge HumanRat Pan Tissue | 2.0572 | 0.6036 | 0.7483 | 63.62% |  |
| SuppFig12O | Heart | DNAmRelativeAge HumanRat Pan Tissue | 0.5471 | 0.1613 | 0.2157 | 60.57% | 56.49% |
| Fig2A | Blood | DNAmAge Pan Tissue | 1.7767 | 0.7497 | 0.8435 | 52.53% |  |
| Fig2E | Blood | DNAmAge Blood | 2.1596 | 0.6614 | 0.7867 | 63.57% |  |
| Fig2I | Blood | DNAmAge HumanRat Pan Tissue | 1.7369 | 0.6152 | 0.4429 | 74.50% |  |
| Fig2M | Blood | DNAmRelativeAge HumanRat Pan Tissue | 0.4724 | 0.1777 | 0.1585 | 66.46% | 64.26% |
| SuppFig12A | Blood | DNAmAge Pan Tissue | 1.9947 | 0.6744 | 0.7830 | 60.75% |  |
| SuppFig12E | Blood | DNAmAge Blood | 1.9523 | 0.6094 | 0.7385 | 62.18% |  |
| SuppFig12I | Blood | DNAmAge HumanRat Pan Tissue | 2.0081 | 0.5998 | 0.4395 | 78.11% |  |
| SuppFig12M | Blood | DNAmRelativeAge HumanRat Pan Tissue | 0.5394 | 0.1630 | 0.1533 | 71.58% | 68.15% |

**Supplementary Table S4: Mean values of the DNAm age estimates of the six epigenetic clocks in the E5 plasma treatment study.** The columns report the figure panels of the corresponding barplot. The tissue that was used to evaluate the clock. The type of clock. The percentage of rejuvenation (last column) was calculated as follows: 100*(1-Old Treated/OldControl). The results for Figure 2 involved the preliminary versions of the six rat clocks. The results for Supplementary Figure 8 involved the final versions of the six rat clocks as detailed in Methods.

According to the 6 epigenetic clocks, the plasma fraction treatment rejuvenated liver by 74.6% (ranging from 68.6% to 78.6% depending on the clock), blood by 64.3% (ranging from 52.5 to 74.5%), heart by 46.5%, and hypothalamus by 24.4%. The rejuvenation effects are even more pronounced if we use the final versions of our epigenetic clocks: liver 77.6%, blood 68.2%, heart 56.5%, hypothalamus 29.6%. According to the final version of the epigenetic clocks, the average rejuvenation across four tissues was 67.40%.

| **Groups** | **Grip Strength in N** | | |
| --- | --- | --- | --- |
|  | **Old control** | **Treatment** | **Adult Control** |
| **0 Day** | 6.10±0.32 | 6.31±0.44 | 10.25±1.01 |
| **4 Day** | 6.24±0.44 | 8.25±0.94 | 10.84±1.12 |
| **8 Day** | 6.01±0.46 | 11.38±0.83 | 10.55±0.99 |
| **15 Day** | 6.00±0.89 | 11.55±0.88 | 11.35±1.15 |
| **30 Day** | 5.78±0.75 | 11.74±0.76 | 12.01±1.23 |

**Supplementary Table S5:** Measurement (with standard deviations) of grip strength of indicated groups of 6 rats each. These average values formed the graphs in Supplementary Figure 10B.

**Histopathological evaluation of rat tissues**

| **Rat group** | **Liver** | **Lungs** | **Kidney** | **Heart** | **Spleen** | **Brian** | **Testes** |
| --- | --- | --- | --- | --- | --- | --- | --- |
| **Old Control** | Granular degeneration of mild severity, minimally multifocal minimal periportal mononuclear cell infiltration | NAD | NAD | NAD | NAD | NAD | NAD |
| **Old Control** | Granular degeneration of mild severity | NAD | NAD | NAD | NAD | NAD | NAD |
| **Old Control** | Granular degeneration of mild severity | NAD | NAD | NAD | NAD | NAD | NAD |
| **Treatment** | Granular degeneration of mild severity | NAD | NAD | NAD | NAD | NAD | NAD |
| **Treatment** | Granular degeneration of mild severity | NAD | NAD | NAD | NAD | NAD | NAD |
| **Treatment** | Granular degeneration of mild severity | NAD | NAD | NAD | NAD | NAD | NAD |
| **Young Control** | Granular degeneration of mild severity | NAD | NAD | NAD | NAD | NAD | NAD |
| **Young Control** | Granular degeneration of mild severity | NAD | NAD | NAD | NAD | NAD | NAD |
| **Young Control** | Granular degeneration of mild severity | NAD | NAD | NAD | NAD | NAD | NAD |

**Supplementary Table S6:** Results of histopathological analyses of rat tissues. Lesions suggestive of any toxicity were not noted. NAD= No Abnormalities Detected. Representative images of the tissues are shown in Supplementary Figure 11.

|  | **Groups** | Hb  (gm %) | RBC  (x 10^6^ /cmm) | WBC  (X 10^3^  /cmm) | Platelets (X 10^5^/ cmm) | HCT (%) | MCV (fl) | MCH (pg) | MCHC g/dl | Lymphocytes (10³ cells/µl) |
| --- | --- | --- | --- | --- | --- | --- | --- | --- | --- | --- |
| 0 Day | Old | 13.10±0.8 | 6.65±0.5 | 10.87±1.0 | 803.00±6.2 | 43.03±1.0 | 54.34±0.9 | 19.29±0.8 | 22.86±0.7 | 6.48±0.8 |
|  | Old treated | 13.26±1.0 | 6.21±0.6 | 10.63±1.2 | 801.83±5.3 | 41.61±2.1 | 53.27±1.5 | 19.13±0.7 | 21.31±1.2 | 6.35±1.1 |
|  | Young | 14.85±0.9 | 5.28±0.9 | 9.40±0.7 | 705.33±8.0 | 40.31±0.6 | 49.83±1.3 | 17.74±1.0 | 18.10±1.1 | 5.17±0.7 |
| 60 Day | Old | 13.46±0.9 | 6.93±0.4 | 10.22±0.4 | 804.17±4.0 | 42.11±1.7 | 55.01±0.9 | 20.65±0.8 | 23.68±1.3 | 6.04±0.3 |
|  | Old treated | 14.52±1.5 | 6.07±0.4 | 9.30±0.6 | 791.67±7.2 | 40.11±1.3 | 51.60±1.2 | 18.02±1.0 | 19.64±1.3 | 5.92±0.7 |
|  | Young | 14.24±0.5 | 5.41±0.4 | 9.17±0.5 | 706.17±7.6 | 40.14±1.1 | 50.40±0.8 | 19.36±0.7 | 19.76±1.3 | 5.51±0.9 |
| 155 Day | Old | 13.56±0.5 | 6.87±0.6 | 10.65±0.5 | 806.50±5.1 | 42.86±0.8 | 54.71±0.9 | 21.77±1.0 | 24.16±0.8 | 6.08±0.2 |
|  | Old treated | 14.26±0.9 | 5.90±06 | 9.16±0.3 | 789.00±3.5 | 40.80±1.4 | 50.63±1.3 | 18.97±0.7 | 18.99±0.8 | 5.72±0.5 |
|  | Young | 14.21±0.7 | 5.55±0.6 | 9.28±0.9 | 709.83±7.7 | 39.88±1.0 | 50.55±0.7 | 19.52±0.7 | 19.81±1.0 | 5.51±0.7 |

**Supplementary Table S7: Blood indices measurements (with standard deviation) at indicated time points post-treatment**. Each measurement was taken from 6 rats per group. These average values formed the graphs in Supplementary Figure 13.

|  | | |  |  |  |
| --- | --- | --- | --- | --- | --- |
| **Sr. No.** | **Parameter** | **Group** | **Old** | **Old Treated** | **Young** |
| **1** | **Total Bilirubin (mg/dL)** | 0 Day | 0.90±0.10 | 0.90±0.11 | 0.56±0.08 |
|  |  | 30 Day | 0.92±0.09 | 0.83±0.12 | 0.55±0.09 |
|  |  | 60 Day | 0.92±0.11 | 0.82±0.10 | 0.57±0.08 |
|  |  | 90 Day | 0.97±0.11 | 0.78±0.09 | 0.57±0.08 |
|  |  | 125 Day | 1.02±0.12 | 0.72±0.09 | 0.59±0.08 |
|  |  | 155 Day | **1.08±0.12** | **0.68±0.07 ###** | **0.60±0.07** |
|  |  |  |  | ***** |  |
| **2** | **Direct Bilirubin (mg/dL)** | 0 Day | 0.575±0.06 | 0.583±0.07 | 0.257±0.05 |
|  |  | 30 Day | 0.588±0.06 | 0.563±0.06 | 0.258±0.05 |
|  |  | 60 Day | 0.605±0.07 | 0.520±0.05 | 0.273±0.04 |
|  |  | 90 Day | 0.618±0.06 | 0.490±0.06 | 0.283±0.04 |
|  |  | 125 Day | 0.640±0.06 | 0.468±0.07 | 0.293±0.05 |
|  |  | 155 Day | **0.680±0.08** | **0.438±0.04 ###** | **0.308±0.04** |
|  |  |  |  | ******* |  |
| **3** | **Glucose (mg/dL)** | 0 Day | 173.0±5.57 | 174.9±4.97 | 152.0±8.80 |
|  |  | 30 Day | 174.4±5.13 | 169.6±2.88 | 152.3±8.34 |
|  |  | 60 Day | 178.7±5.02 | 167.5±3.53 | 155.4±8.69 |
|  |  | 90 Day | 180.0±5.37 | 165.3±3.00 | 157.9±9.60 |
|  |  | 125 Day | 183.2±5.04 | 163.5±3.59 | 161.2±9.55 |
|  |  | 155 Day | **186.0±3.32** | **163.6±4.10 ###** | **164.6±10.09** |
|  |  |  |  | ***** |  |
| **4** | **Triglyceride (mg/dL)** | 0 Day | 57.2±10.16 | 56.4±10.45 | 25.0±9.01 |
|  |  | 30 Day | 70.5±5.45 | 45.1±6.67 | 28.9±8.80 |
|  |  | 60 Day | 79.3±5.52 | 45.4±5.84 | 30.8±7.83 |
|  |  | 90 Day | 84.1±5.63 | 43.6±6.05 | 33.1±7.25 |
|  |  | 125 Day | 91.4±5.08 | 41.7±5.78 | 34.7±7.92 |
|  |  | 155 Day | **103.7±5.30** | **38.0±5.64 ###** | **37.9±8.26** |
| **5** | **HDL (mg/dL)** | 0 Day | 109.7±9.19 | 111.3±8.95 | 142.5±6.71 |
|  |  | 30 Day | 110.7±7.05 | 117.2±8.44 | 144.3±5.92 |
|  |  | 60 Day | 108.9±6.89 | 127.0±8.16 | 145.1±6.25 |
|  |  | 90 Day | 108.0±7.67 | 130.6±9.13 | 147.2±6.70 |
|  |  | 125 Day | 105.9±7.78 | 138.2±10.11 | 149.0±6.49 |
|  |  | 155 Day | **102.3±7.24** | **147.2±8.58 ##**** | **151.6±6.68** |
|  |  |  |  |  |  |
| **6** | **Cholesterol (mg/dL)** | 0 Day | 46.8±6.74 | 45.9±6.85 | 17.3±3.17 |
|  |  | 30 Day | 48.1±6.49 | 40.3±6.76 | 17.8±4.21 |
|  |  | 60 Day | 49.1±6.40 | 37.6±5.88 | 18.4±4.33 |
|  |  | 90 Day | 50.4±5.76 | 34.9±7.13 | 19.2±4.12 |
|  |  | 125 Day | 53.7±5.76 | 32.0±6.57 | 21.0±3.78 |
|  |  | 155 Day | **56.6±5.78** | **28.1±5.45 ###** | **23.0±3.75** |
|  |  |  |  | ****** |  |
| **7** | **Creatinine (mg/dL)** | 0 Day | 1.08±0.10 | 1.07±0.12 | 0.29±0.02 |
|  |  | 30 Day | 1.06±0.09 | 1.02±0.13 | 0.35±0.04 |
|  |  | 60 Day | 1.31±0.24 | 0.87±0.11 | 0.39±0.03 |
|  |  | 90 Day | 1.54±0.26 | 0.80±0.10 | 0.44±0.04 |
|  |  | 125 Day | 1.78±0.22 | 0.70±0.08 | 0.50±0.03 |
|  |  | 155 Day | **2.03±0.33** | **0.63±0.08 ###** | **0.54±0.02** |
|  |  |  |  | ***** |  |
| **8** | **BUN (mg/dL)** | 0 Day | 16.23±1.21 | 16.19±0.92 | 4.03±0.13 |
|  |  | 30 Day | 16.36±1.17 | 15.26±0.74 | 4.07±0.15 |
|  |  | 60 Day | 16.66±1.14 | 14.57±0.66 | 4.14±0.15 |
|  |  | 90 Day | 16.80±1.17 | 13.27±0.78 | 4.22±0.15 |
|  |  | 125 Day | 16.99±1.20 | 11.05±0.79 | 4.32±0.16 |
|  |  | 155 Day | **17.11±1.22** | **8.94±0.73 ###** | **4.46±0.15** |
|  |  |  |  | ******* |  |
| **9** | **SGPT (IU/L)** | 0 Day | 34.30±1.65 | 34.12±1.91 | 22.20±1.48 |
|  |  | 30 Day | 34.06±1.42 | 32.20±1.52 | 23.25±1.52 |
|  |  | 60 Day | 34.93±1.21 | 30.78±1.41 | 24.56±1.43 |
|  |  | 90 Day | 36.02±1.18 | 29.78±1.03 | 25.70±1.78 |
|  |  | 125 Day | 37.16±1.11 | 28.87±0.89 | 27.05±1.84 |
|  |  | 155 Day | **38.29±1.23** | **28.03±0.76 ###** | **27.56±1.52** |
|  |  |  |  | ***** |  |
| **10** | **SGOT (IU/L)** | 0 Day | 86.24±3.77 | 86.79±2.35 | 41.53±1.93 |
|  |  | 30 Day | 90.02±3.95 | 80.74±2.15 | 44.86±1.87 |
|  |  | 60 Day | 92.41±3.69 | 75.26±2.28 | 45.39±1.78 |
|  |  | 90 Day | 94.97±3.87 | 66.39±3.17 | 47.55±2.41 |
|  |  | 125 Day | 96.18±3.33 | 60.60±1.22 | 50.39±2.36 |
|  |  | 155 Day | **97.45±2.38** | **54.13±1.85 ###** | **53.54±2.43** |
|  |  |  |  | ****** |  |
| **11** | **Total protein (g/dl)** | 0 Day | 7.59±1.06 | 7.75±0.88 | 4.74±0.95 |
|  |  | 30 Day | 8.22±1.07 | 7.55±0.78 | 5.15±0.73 |
|  |  | 60 Day | 8.73±0.73 | 7.39±0.80 | 5.57±0.77 |
|  |  | 90 Day | 9.83±0.59 | 7.15±0.94 | 5.81±0.87 |
|  |  | 125 Day | 10.96±0.35 | 7.04±0.88 | 6.56±0.80 |
|  |  | 155 Day | **12.14±0.53** | **7.12±0.86 ##** | **7.01±0.86** |

**Supplementary Table S8:** Detailed vital organ biomarker measurements of rats at stated time points post-plasma fraction treatment. The measurements (with standard deviation) were from 6 rats per group. These average values formed the graphs in **Figure 3**.

| **GSH** | **Brain** | **Heart** | **Lung** | **Liver** |
| --- | --- | --- | --- | --- |
| Old Control | 18.55 ± 5.44 | 25.20 ± 15.63 | 13.96 ± 7.55 | 15.03 ± 5.58 |
| Old Treated | 38.67 ± 17.06 | 62.92 ± 18.17 | 33.85 ± 10.80 | 54.02 ± 11.19 |
| Young Control | 41.09 ± 10.77 | 68.38 ± 15.83 | 36.20 ± 18.21 | 60.86 ± 9.72 |

| **MDA** | **Brain** | **Heart** | **Lung** | **Liver** |  |
| --- | --- | --- | --- | --- | --- |
| Old Control | 43.62 ± 8.91 | 77.99 ± 24.19 | 64.61 ± 18.14 | 119.39 ± 24.73 |  |
| Old Treated | 21.11 ± 9.79 | 17.79 ± 9.11 | 21.62 ± 10.91 | 31.86 ± 9.60 |  |
| Young Control | 18.36 ± 8.19 | 16.33 ± 10.16 | 14.49 ± 7.66 | 22.93 ± 10.67 |  |
|  | | | | |  |
| **SOD** | **Brain** | **Heart** | **Lung** | **Liver** | |
| Old Control | 14.65 ± 6.12 | 22.62 ± 9.97 | 19.34 ± 5.39 | 21.53 ± 7.03 | |
| Old Treated | 38.48 ± 6.87 | 51.60 ± 10.46 | 34.29 ± 3.98 | 41.81 ± 3.26 | |
| Young Control | 39.14 ± 4.85 | 53.59 ± 12.14 | 42.64 ± 6.09 | 44.13 ± 3.06 | |

| **Catalase** | **Brain** | **Heart** | **Lung** | **Liver** |
| --- | --- | --- | --- | --- |
| Old Control | 17.93 ± 2.15 | 10.72 ± 3.40 | 8.83 ± 2.03 | 12.14 ± 1.52 |
| Old Treated | 19.73 ± 2.91 | 18.88 ± 5.12 | 13.35 ± 2.45 | 21.04 ± 1.07 |
| Young Control | 21.68 ± 1.87 | 19.27 ± 1.32 | 17.33 ± 1.61 | 22.68 ± 1.08 |

(n=6, Mean ± SD)

**Supplementary Table S9:** Oxidative Stress Parameters. The measurements (with standard deviation) were from 6 rats per group.

| **Group** | **Old Control** | **Old Control (Std Deviation)** | **Treatment** | **Treatment (Std Deviation)** | **Young Control** | **Young Control (Std Deviation)** |
| --- | --- | --- | --- | --- | --- | --- |
| **First Dosing (4 Injection)** | | | | | | |
| **0 Day** | 67.60 | 10.11 | 65.54 | 13.37 | 35.04 | 7.98 |
| **4 Day** | 67.80 | 10.02 | 59.90 | 12.54 | 35.16 | 8.04 |
| **8 Day** | 68.02 | 10.05 | 32.28 | 7.34 | 35.42 | 7.88 |
| **15 Day** | 68.47 | 9.96 | 32.42 | 8.39 | 35.71 | 8.12 |
| **30 Day** | 69.62 | 9.79 | 35.58 | 8.12 | 36.45 | 8.03 |
| **45 Day** | 70.71 | 9.55 | 38.69 | 12.42 | 36.88 | 8.06 |
| **52 Day** | 71.41 | 9.43 | 42.98 | 14.38 | 37.18 | 8.03 |
| **60 Day** | 71.98 | 9.36 | 46.67 | 10.32 | 37.58 | 8.00 |
| **75 Day** | 73.91 | 10.96 | 48.49 | 10.42 | 38.02 | 8.03 |
| **90 Day** | 75.04 | 10.91 | 49.50 | 10.43 | 38.81 | 8.10 |
| **Second Dosing (4 Injection)** | | | | | | |
| **95 Day (0 Day)** | 75.16 | 10.96 | 49.62 | 10.59 | 38.63 | 8.18 |
| **99 Day (4 Day)** | 75.28 | 10.92 | 44.03 | 10.20 | 38.79 | 8.16 |
| **103 Day (8 Day)** | 75.40 | 10.79 | 34.86 | 10.65 | 38.77 | 8.24 |
| **110 Day (15 Day)** | 75.48 | 10.90 | 33.61 | 10.25 | 38.65 | 8.32 |
| **125 Day (30 Day)** | 76.53 | 10.83 | 32.50 | 10.24 | 39.01 | 8.29 |
| **140 Day (45 Day)** | 79.25 | 11.12 | 33.23 | 10.33 | 41.01 | \| 9.05 \| \| --- \| |
| **155 Day (60 Day)** | 83.04 | 10.81 | 36.03 | 9.31 | 42.90 | 9.17 |

**Supplementary Table S10. IL6 measurements**. The measurements (with standard deviation) were from 6 rats per group.


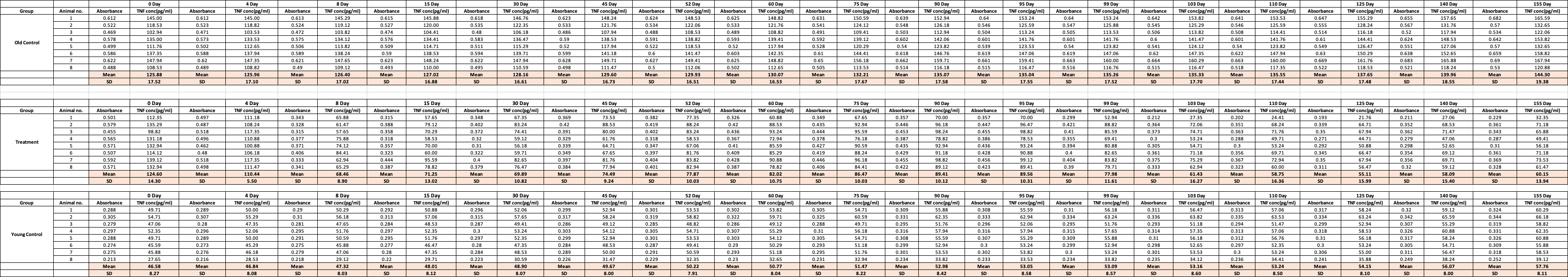


**Supplementary Table S11. TNF-alpha measurements**.


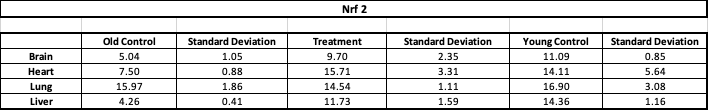


**Supplementary Table S12. Nrf2 measurements in different rat tissues.**

| Glycoform | Treatment effect | Treatment standard error | *p*-value | Adjusted *p*-value |
| --- | --- | --- | --- | --- |
| IgG2b-H3N5F1 | 1.969 | 0.460 | 8.29E-05 | 2.16E-03 |
| IgG2a-H4N4F1 | 1.484 | 0.385 | 3.20E-04 | 4.16E-03 |
| IgG2a-H3N4F1 | -1.561 | 0.421 | 5.10E-04 | 4.42E-03 |
| IgG2a-H5N4F1 | 1.606 | 0.452 | 8.11E-04 | 5.27E-03 |
| IgG2b-H5N5F1 | -1.339 | 0.419 | 2.34E-03 | 1.22E-02 |
| IgG2b-H4N4F1 | 0.841 | 0.303 | 7.49E-03 | 2.85E-02 |
| IgG2c-H3N5F1 | -1.137 | 0.411 | 7.66E-03 | 2.85E-02 |
| IgG2b-H3N3F1 | -1.290 | 0.496 | 1.18E-02 | 3.84E-02 |
| IgG2c-H5N4F1G1 | -1.182 | 0.473 | 1.54E-02 | 4.46E-02 |
| IgG2b-H3N4F1 | -0.802 | 0.341 | 2.21E-02 | 5.75E-02 |
| IgG2a-H5N4F1G1 | 1.106 | 0.475 | 2.65E-02 | 6.13E-02 |
| IgG2a-H4N5F1G1 | 1.097 | 0.477 | 2.83E-02 | 6.13E-02 |
| IgG2c-H3N4 | -0.958 | 0.462 | 4.23E-02 | 8.45E-02 |
| IgG2c-H3N4F1 | 0.970 | 0.480 | 4.79E-02 | 8.89E-02 |
| IgG2b-H4N5F1G1 | 0.856 | 0.444 | 5.84E-02 | 9.66E-02 |
| IgG2b-H5N4F1G1 | 0.691 | 0.353 | 5.95E-02 | 9.66E-02 |
| IgG2a-H3N5F1 | 0.788 | 0.442 | 7.97E-02 | 1.22E-01 |
| IgG2a-H4N4F1G1 | 0.922 | 0.536 | 9.01E-02 | 1.30E-01 |
| IgG2c-H4N4F1G1 | -0.777 | 0.494 | 1.20E-01 | 1.64E-01 |
| IgG2b-H5N4F1 | -0.534 | 0.379 | 1.67E-01 | 2.17E-01 |
| IgG2a-H8N2 | 0.503 | 0.531 | 3.46E-01 | 4.28E-01 |
| IgG2c-H3N3F1 | 0.348 | 0.383 | 3.67E-01 | 4.34E-01 |
| IgG2c-H4N4F1 | 0.399 | 0.468 | 3.95E-01 | 4.47E-01 |
| IgG2a-H3N4 | 0.389 | 0.527 | 4.62E-01 | 5.00E-01 |
| IgG2a-H3N3F1 | -0.215 | 0.484 | 6.58E-01 | 6.84E-01 |
| IgG2b-H4N4F1G1 | 0.141 | 0.448 | 7.53E-01 | 7.53E-01 |

**Supplementary Table S13. Glycan age analysis in rat blood.** Plasma fraction E5 treatment effect on rat IgG2a, 2b, and 2c N-glycoform composition. Glycoproteomic data was analyzed using a linear mixed-effects model and p-values < 0.05 were considered statistically significant. The rows correspond to different rat IgG tryptic peptides as detailed in the following panel.

**Rat IgG tryptic peptides and UniProt accesion numbers:**

IgG2a – P20760, QSNSTLR

IgG2b – P20761, EEQYNSTFR

IgG2c – P20762, VFTAQTQPHEEQLNGTFR

N – glycosylation site

**Glycan composition:**

H – hexose

N – *N*-acetylglucosamine

F – fucose

G – *N*-glycolylneuraminic acid

**Methods: Gel Electrophoresis study for extracted RNAs from E5**

Below, we outline the process of identifying both low and high molecular weight RNA in the E5 sample.

RNA Isolation Procedure:

Combine 400 μl of E5 solution with 500 μl of TRIzol reagent in a 1.5 ml Eppendorf tube. Ensure thorough mixing using a tube shaker.

Introduce 100 μl of chloroform to the mixture. Vigorously shake the tube 15-20 times and let it stand at room temperature (RT) for 2-5 minutes. Subsequently, centrifuge the mixture at 12,000 rpm for 15 minutes at 4°C.

Carefully collect the clear supernatant from the previous step and mix it with an equal volume of isopropanol (IPA). Centrifuge this at 10,000 rpm for 5 minutes at 4°C.

Remove the supernatant and resuspend the pellet in 500 μl of 70% ethanol. Centrifuge again at 10,000 rpm for 5 minutes at 4°C.

Allow the pellet to air dry partially, ensuring it remains slightly moist.

Resuspend the pellet in approximately 10 μl of DEPC water and store at -20°C.

Gel Electrophoresis Methodology:

1. Gel Preparation:

Prepare a 2% w/w agarose gel in 1X TAE buffer. Dissolve 2g of agarose in 100 ml of buffer, heat until clear, and add 3 μl of Ethidium bromide. Pour into a tray, insert a comb to form wells, and let it solidify.

2. Sample Preparation:

Dilute RNA samples at ratios of 1:10, 1:20, and 1:50 using DEPC water. Mix each RNA sample (10 μl) with 2 μl of loading dye.

3. Gel Electrophoresis Execution:

Place the solidified gel in an electrophoresis chamber filled with 1X TAE buffer. Load the samples and a 3 μl molecular ladder into the wells. Connect the power supply, set the voltage to 70, and run the electrophoresis.

4. Visualization:

After completion, remove the gel and visualize the bands using a Gel documentation system.

## **Protocol for Western Blotting of CD63 and CD81**

Expression of CD63 (Molecular Weight: 53 kDa)

and CD81 (Molecular Weight: 23 kDa) in E5 Fractions was determined using CD 63 (1:1000) and CD 81 (Rabbit 1:1000) (ABclonal Technology, USA) primary antibody and peroxidase-labeled goat anti-rabbit IgG secondary antibody.

*Sample Lysate Preparation*

A small amount of E5 fraction (500 µl) was mixed with ice-cold radio-immuno-precipitation assay (RIPA) buffer (500 µl)) for 2 hours. After 2 hours, the E5 fractions were centrifuged at 8000 RPM for 10 minutes. The supernatant was used for the determination of total protein content in the E5 fraction. Total protein was estimated by using the Bradford method.

*Preparation of sample for loading*

Samples from different E5 fractions were incubated with 4X Lamelli sample buffer (5µl Laemmli) at 95℃ for 5 minutes in the dry bath for protein denaturation. Samples were further centrifuged at 1000 rpm for 1 minute. 20 µg samples were loaded in each well.

*Running buffer (pH 8.3)*

Running buffer was prepared by dissolving 3.02 g of tris base (25 mM), 14.26 g of glycine (190 mM), and 1 g of sodium dodecyl sulphate (10% w/v) in 800 ml of water. pH was adjusted to 8.3 with conc. HCl and final volume was made up to 1000 ml with distilled water.

*Transfer buffer*

Running buffer was prepared by dissolving 3.02 g of tris base (25 mM), 14.26 g of glycine (190 mM), and 200 ml of methanol (20 %) in 800 ml of water. The final volume was made up to 1000 ml with distilled water.

*Sodium dodecyl sulfate-polyacrylamide gel electrophoresis (SDS-PAGE)*

10 µL of samples were loaded and protein separation was carried out on 8 % SDS-PAGE Gel at a constant current of 50V for an initial 15 minutes and 110 V for approximately 90 minutes using tris glycine buffer. The preparation methods of resolving gel and stacking gel are as follows.

*Method of preparation of 8 % acrylamide-resolving gel*

| Ingredients | Quantity for 10 ml |
| --- | --- |
| Double distilled water | 4.6 mL |
| 30% Acrylamide Stock Solution | 2.7 mL |
| 1.5 M Tris(pH=8.8) | 2.5 mL |
| 10% (w/v) sodium dodecyl sulphate | 0.1 mL |
| 10% (w/v) ammonium persulfate (APS)* | 0.1 mL |
| Tetramethylethylenediamine (TEMED)* | 8 µL |

*APS and TEMED were added right before use.

*Method of preparation of stacking gel*

| Ingredients | Quantity for 5 ml |
| --- | --- |
| Double distilled water | 3.4 ml |
| 0.5 M Tris-HCl, pH 6.8 | 0.63 ml |
| 10% (w/v) sodium dodecyl sulphate | 50 µl |
| 30% Acrylamide Stock Solution | 0.830 ml |
| 10% (w/v) ammonium persulfate (APS)* | 50 µl |
| Tetramethylethylenediamine (TEMED)* | 5 µl |

*APS and TEMED was added right before use.

*Protein transfer (Blotting)*

The protein transfer (Blotting) was done on Polyvinylidene fluoride (PVDF) membrane using a transfer buffer. The transfer was carried out using the Trans-Blot® SD semi-dry transfer cell (Bio-Rad). Bands were visualized on the membrane with a Ponceau stain. A membrane was blocked with 5% w/v non-fat dried milk powder in Tris buffer saline (TBS) solution for 2 hours.

*Incubation with primary and secondary antibody*

Membranes were separately incubated with primary antibodies (CD 63 and CD 81) at dilution 1:1000 and 1:1000 in TBS-T with constant shaking overnight at 4 °C. Three washings of 15 minutes each were given to the membrane with TBS-T to remove excess primary antibodies. Membranes were further incubated with secondary antibody at dilution 1:2000 with constant shaking for 1.5 hours. Three washings were performed for 15 minutes each with TBS-T. The blots were visualized using Bio-Rad Molecular Imager® ChemiDoc XRS+ System with Image Lab™ Software by Bio-Rad.

**Technical Details and software for the rat clocks**

The epigenetic clock software for rats can be applied to data generated on the mammalian array platform ^1^. New data for epigenetic clock studies can be generated with the mammalian methylation array (HorvathMammalMethylChip40), which is distributed by the Epigenetic Clock Development Foundation: https://clockfoundation.org/

The coefficient values and CpGs underlying the clocks can be found in a Supplementary Table.

**Statistical methods used for building the clocks**

The epigenetic clocks were used by employing a single elastic net regression model analysis (R function glmnet). We use used Leave-one-out analysis (LOO) using a single lambda value. We chose the following parameters for the glmnet R function (Alpha: 0.5, CV Fold: 10, Lambda choice for Clock: 1 standard error above minimum CV-MSE).

We report 2 sets of clocks (preliminary versions and final versions). We recommend to use the final versions. We only report the preliminary versions so that readers can recreate Figure 1 and Figure 2 in our article.

**Clocks**

1. The preliminary and final version of the pan tissue clock for rats

is based on 195 CpGs and 193 CpGs which are specified in columns

"Coef.PrelimRatPanTissue" and "Coef.FinalRatPanTissue", respectively. No age transformation was carried out.

1. The preliminary and final version of the blood tissue clock for rats

is based on 48 CpGs and 51 CpGs which are specified in columns

"Coef.PrelimRatBloodTissue" and "Coef.FinalRatBloodTissue", respectively. No age transformation was carried out.

1. The preliminary and final version of the Brain tissue clock for rats

is based on 99 CpGs and 108 CpGs which are specified in columns

"Coef.PrelimRatBrainTissue" and "Coef.FinalRatBrainTissue", respectively. No age transformation was carried out.

1. The preliminary and final version of the Liver tissue clock for rats

is based on 62 CpGs and 46 CpGs which are specified in columns

"Coef.PrelimRatLiverTissue" and "Coef.FinalRatLiverTissue", respectively. No age transformation was carried out.

1. Preliminary and final versions of the human rat pan tissue clock are specified in Coef.PrelimHumanRatPanTissueLogLinearAge (700 CpGs) and Coef.FinalHumanRatPanTissueLogLinearAge (989 CpGs). Note that a log linear transformation was applied.
2. Preliminary and final versions of the human rat blood tissue clock are specified in Coef.PrelimHumanRatBloodTissueLogLinearAge (97 CpGs) and Coef.FinalHumanRatBloodTissueLogLinearAge (72 CpGs). Note that a log linear transformation was applied.
3. Preliminary and final versions of the human rat pan tissue clock for RELATIVE AGE are specified in Coef.PrelimHumanRatRelativeAgePan (870 CpGs) and Coef.FinalHumanRatRelativeAgePan (738 CpGs). Note that we define RelativeAge=Age/maxLifespan where maxLifespan is 122.5 for humans and 3.8 years for rats, respectively. Age is in units of years.
4. Preliminary and final versions of the human rat blood tissue clock for RELATIVE AGE are specified in Coef.PrelimHumanRatRelativeAgeBlood (138 CpGs) and Coef.FinalHumanRatRelativeAgeBlood (145 CpGs).

The DNAm Age estimate is estimated as follows. Form a weighted linear combination of the CpGs whose details can be found in the Supplementary File, SupplementaryData.RatClockCoef.csv.

This will result in a number referred to as LinearCombination. Some of the clocks involve a log linear transformation whose inverse needs to be applied.

The Supplementary file reports the probe identifier (cg number) used in the custom Infinium array (HorvathMammalMethylChip40). The weights used in this linear combination are specified in the respective column entitled "Coef.".

The formula assumes that the DNA methylation data measure "beta" values, but the formula could be adapted to other ways of generating DNA methylation data.

### General description of age transformation

The human-rat clocks for chronological age used log linear transformations that are similar to those employed for the HUMAN pan tissue (Horvath 2013, Genome Biology).

An elastic net regression model (implemented in the glmnet R function) was used to regress a transformed version of age on the beta values in the training data. The glmnet function requires the user to specify two parameters (alpha and beta). Since we used an elastic net predictor, alpha was set to 0.5. But the lambda value of was chosen by applying a 10 fold cross validation to the training data (via the R function cv.glmnet).

The elastic net regression results in a linear regression model whose coefficients b_0_, b_1_, . . . , relate to transformed age as follows
*F*(chronological age)=*b*_0_*+b*_1_*CpG*_1_*+ . . . +b*_p_*CpG*_p_+error

Note that the intercept term is denoted by b_0_. The coefficient values can be found in the Supplementary file. Based on the coefficient values from the regression model, DNAmAge is estimated as follows

*DNAm*Age=$F^{-1}$(*b*_0_*+b*_1_*CpG*_1_*+ . . . +b*_p_*CpG*_p_)

, where $F^{-1}\left( y \right)$ denotes the mathematical inverse of the function F(.). Thus, the regression model can be used to predict to transformed age value by simply plugging the beta values of the selected CpGs into the formula.

### Defining Properties of the log linear transformation

As indicated by its name, the “log-linear” function, has a logarithmic dependence on age before the average age of sexual maturity (of the species) and a linear dependence after Age at Sexual Maturity (of the species). For the human-rat clocks we used the following averages at sexual maturity (in units of years): 13.5 years for humans and 0.219178082 years for rats (**Supplementary Table**).

We used a piecewise transformation, parameterized by Age of Sexual Maturity ($A$).

The transformation is F(x), given by

$$F\left( x \right)=g\left( \frac{x+1.5}{A+1.5} \right)\text{ where }g\left( t \right)= \left\{ \begin{aligned} \begin{aligned} \begin{aligned} \log\left( t \right), for 0\leq t\leq1 \\ t-1, for 1\leq t \end{aligned} \end{aligned} \end{aligned} \right.$$

Explicitly, F(x) is given by

$$F\left( x \right)=\left\{ \begin{aligned} \begin{aligned} \begin{aligned} \log\left( \frac{x+1.5}{A+1.5} \right), for 0\leq x\leq A \\ \frac{x-A}{A+1.5}, for A\leq x \end{aligned} \end{aligned} \end{aligned} \right.$$

In order to use this transformation to predict Age on *new samples*, one needs to use the *inverse* transformation, F^-1^(y), given by

$$F^{-1}\left( y \right)= \left\{ \begin{aligned} \begin{aligned} \begin{aligned} \left( A+1.5 \right)*\text{exp}\left( y \right)-1.5, for y\leq0 \\ (A+1.5)y+A, for y\geq0 \end{aligned} \end{aligned} \end{aligned} \right.$$

For predicting age, apply the inverse transformation to coefficient-weighted sum. That is,

$$DNAmAge=F^{-1}\left( x*\beta\right)$$

where $\beta$ is the vector of coefficients and $x$ is the vector of methylation values, with an intercept term.

### R Implementation of the log linear transformation

### Applies the log linear transformation to the input vector x, i.e. to Age

F= Vectorize(function(x, maturity, ...) {

if (is.na(x) | is.na(maturity)) {return(NA)}

k <- 1.5

y <- 0

if (x < maturity) {y = log((x+k)/(maturity+k))}

else {y = (x-maturity)/(maturity+k)}

return(y)

})

### Inverse log linear transformation

F.inverse= Vectorize(function(y, maturity, ...) {

if (is.na(y) | is.na(maturity)) {return(NA)}

k <- 1.5

x <- 0

if (y < 0) {x = (maturity+k)*exp(y)-k}

else {x = (maturity+k)*y+maturity}

return(x)

})

### The DNAm Age estimate is estimated in two steps.

First, one forms a weighted linear combination of the CpGs whose details can be found in Table

The table reports the probe identifier (cg number) used in the custom Infinium array (HorvathMammalMethylChip40). The weights used in this linear combination are specified in the respective column entitled "Coef.".

The formula assumes that the DNA methylation data measure "beta" values but the formula could be adapted to other ways of generating DNA methylation data.

**Pseudo R code**

# R function for multivariate regression model

multivariatePredictorCoef=function(dat0, datCOEF,imputeValues=FALSE) {

datout=data.frame(matrix(NA,nrow=dim(dat0)[[2]]-1,ncol=dim(datCOEF)[[2]]-1 ))

match1=match(datCOEF[-1,1],dat0[,1] )

if ( sum(!is.na(match1))==0 ) stop("Input error. The first column of dat0 does not contain CpG identifiers (cg numbers).")

dat1=dat0[match1,]

row.names1=as.character(dat1[,1])

dat1=dat1[,-1]

if (imputeValues ){dat1=impute.knn(data=as.matrix(dat1) ,k = 10)[[1]]}

for (i in 1:dim(dat1)[[2]] ){ for (j in 2:dim(as.matrix(datCOEF))[[2]] ){

datout[i,j-1]=sum(dat1[,i]* datCOEF[-1,j],na.rm=TRUE)+ datCOEF[1,j]}}

colnames(datout)=colnames(datCOEF)[-1]

rownames(datout)=colnames(dat0)[-1]

datout=data.frame(SampleID= colnames(dat0)[-1],datout)

datout

} # end of function

# read in supplementary table

datCoef=read.csv("TableS.csv")

The first columns should read as follows

names(datCoef)

1. var
2. Coef.PrelimRatPanTissue
3. Coef.PrelimRatBloodTissue
4. Coef.PrelimRatBrainTissue
5. Coef.PrelimRatLiverTissue
6. Coef.PrelimHumanRatPanTissueLogLinearAge
7. Coef.PrelimHumanRatBloodTissueLogLinearAge
8. Coef.PrelimHumanRatRelativeAgePan
9. Coef.PrelimHumanRatRelativeAgeBlood
10. Coef.FinalRatPanTissue
11. Coef.FinalRatBloodTissue
12. Coef.FinalRatBrainTissue
13. Coef.FinalRatLiverTissue
14. Coef.FinalHumanRatPanTissueLogLinearAge
15. Coef.FinalHumanRatBloodTissueLogLinearAge
16. Coef.FinalHumanRatRelativeAgePan
17. Coef.FinalHumanRatRelativeAgeBlood

ETC ETC

# Restrict attention to the FINAL rat clocks columns

datCoef=datCoef[,c(1,10:17)]

# assume the first column of dat0 contains the CpG identifiers

match1=match(datCoef[-1,1],dat0[,1] )

missingProbes= as.character(datCoef[-1,1] )[is.na(match1)]

dat1=dat0[match1,]

# data frame with predicted values.

datPredictions=multivariatePredictorCoef(dat1,datCOEF=datCoef,imputeValues=FALSE)

#let's relabel the columns by replacing "Coef" with "DNAm" since the columns contain estimates of age or relative age instead of coefficient values

colnames(datPredictions)=gsub(pattern="Coef", replacement="DNAm", x=colnames(datPredictions))

# We need to transform the human rat clock for chronological age using the inverse of the log linear #transformation.

For rats, the age at sexual maturity has to be set to 0.21917808 years.

datPredictions$DNAm.FinalHumanRatPanTissueLogLinearAge= F.inverse(datPredictions$DNAm.FinalHumanRatPanTissueLogLinearAge, maturity=0.21917808)

datPredictions$DNAm.FinalHumanRatBloodTissueLogLinearAge= F.inverse(datPredictions$DNAm.FinalHumanRatBloodTissueLogLinearAge, maturity=0.21917808)

#The data frame "datPredictions" contains the age estimates in units of years and relative age estimates.

1 Arneson, A. *et al.* A mammalian methylation array for profiling methylation levels at conserved sequences. *Nature Communications* **13**, 783 (2022). <https://doi.org:10.1038/s41467-022-28355-z>
